# Stability analysis of hydrodynamic journal bearings with variable axial geometrical configuration using titanium dioxide nanoparticles as lubricant additives

**DOI:** 10.1038/s41598-026-47711-3

**Published:** 2026-04-24

**Authors:** H. Awad, E. Saber, Khaled M. Abdou, S. Dajani

**Affiliations:** Arab Academy for Science, Technology and Maritime Transport (AASTMT), Abu-Kir, Alexandria, Egypt

**Keywords:** Journal bearings, Nanolubricant, Dynamic behavior of bearings, Stability analysis, Nanoparticles, Aggregate particle size, Engineering, Materials science, Mathematics and computing, Nanoscience and technology, Physics

## Abstract

The dynamic stability of high-speed rotor systems is highly dependent on the interaction between bearing geometry and lubricant rheology. The present work examines the stability of hydrodynamic journal bearings with four different axial configurations: conical (wedge), concave, convex, and wavy, lubricated with TiO₂ nanofluids. The integration of a modified Krieger-Dougherty viscosity model that takes into consideration packing fractions and nanoparticle aggregation, extending beyond conventional static volume-fraction assumptions, is an important advance of this work. The governing Reynolds-like equation is expressed in curvilinear coordinates and numerically solved to determine the transition between stable and unstable vibrations. The results show that nanoparticle aggregate size and packing percent strongly influence the critical stability number, with larger aggregation generally improving the stable working range. The stability limitations are studied using a numerical simulation of the journal’s vibration behavior. Vibration data is collected to create a stability map, which represents the transition between stable and unstable vibrations as a function of eccentricity ratio and stability number. It is found that concave and wedge geometries outperform the other shapes investigated; but the concave geometry is recommended to attain the highest critical stability number for a given eccentricity ratio.

**Supplementary Information:**

The online version contains supplementary material available at 10.1038/s41598-026-47711-3.

## Introduction

### Foundations of journal bearing stability

The stability analysis of journal bearings rotating at high speeds is of critical importance to modern rotor dynamics. Due to the inherent relationship between fluid film dynamics and rotor performance, researchers have explored various factors influencing system behavior. Kumar and Mishra^1^ numerically investigated the impact of geometric changes due to wear following turbulent lubrication theory. Rameshet et al.^2^ examined the effect of surface roughness on the stability of submerged oil elliptical journal bearings under dynamic loads using an average flow model. Furthermore, Kakoty and Majumdar^3^ utilized a linear perturbation approach to study fluid film inertia on the stability of bearings mounted on flexible supports, finding that inertia effects are vital for reliable predictions in high-speed utilities.

### Rheological considerations and computational modeling

Advanced lubrication research has expanded to address complex rheological behaviors and modeling techniques. Raghunandana and Majumdar^4^ investigated the influence of non-Newtonian lubricant behavior caused by polymers on bearing stability. Similarly, Weng and Chen^5^ studied linear stability by accounting for surface roughness and flow rheology, linearizing the modified Reynolds equation and rotor motion equations around an equilibrium location. The effects of wear on the dynamic behavior of flexible rotors supported by powder-lubricated bearings were explored by Rahmani et al.^6^. To facilitate these analyses, Hu et al.^7^ developed modeling software using Matlab and Simulink to simulate and identify dynamic behaviors in rotors supported by multiple hydrodynamic bearings.

### Nanoparticles as lubricant additives

The integration of nanoparticles as lubricant additives represents a significant advancement in enhancing bearing performance. Beyond improving lubrication, nanoparticles can be used to coat friction surfaces with a protective layer^8^. These additive depositions have been shown to compensate for material loss through indirect effects^9^ and effectively lower friction surface roughness^10^. While nanofluids exhibit higher effective viscosity than conventional base fluids—determined primarily by concentration and size—there remains a scarcity of data regarding their impact on dynamic stability. Although many classical models for nanofluid viscosity have been established in the literature^11–19^, most research has concentrated on the steady-state properties of bearings operating with various nanoparticle additives^20–25^.

### Impact of couple stress fluids on dynamic stability

The properties of journal bearings lubricated by couple stress fluids have also been extensively documented^26–33^. Findings generally suggest that utilizing couple stress lubricants improves load-carrying capacity and reduces the coefficient of friction. Senator et al.^34^ investigated the effect of couple stress fluid characteristics on film forces specifically during unstable operation situations. Mehta et al.^35^ utilized a finite element approach to study the stability of two-lobe hydrodynamic bearings, revealing that the couple stress parameter significantly impacts stiffness and damping coefficients while increasing the stability threshold speed. Additionally, Kumar et al.^36^ analyzed the dynamic behavior of spindle motion in lathe machines, solving non-linear equations of motion via the Runge–Kutta approach to confirm that couple stress fluids enhance overall rotor stability.

### Previous findings on misalignment and axial geometry

Foundational research by Hamed and Saber^37^ utilized perturbation techniques and numerical simulations of vibration behavior to evaluate rotor stability under dynamic conditions. Complementary studies by Saber and Abdou^38^ provided a thorough investigation into the dynamic stability and responses of misaligned fluid film bearings. Recently, Awad et al.^39^ specifically examined the impact of titanium dioxide (TiO₂) nanoparticle volume percentages and aggregate sizes on the steady-state and stability constraints of plain journal bearings. Subsequent work^40^ investigated the influence of axial geometrical arrangements—including conical (wedge), concave, convex, and wavy surfaces—on steady-state characteristics. This research established that modifying the bearing’s axial shape increases load-carrying capacity and decreases friction compared to standard cylindrical designs, with concave geometry demonstrating clear superiority.

### Contemporary developments in rotor system dynamics

While the current study focuses on the localized fluid film dynamics and stability of specifically profiled journal bearings, it is important to situate these findings within the broader context of complex rotor system assemblies. Recent literature in rotor dynamics has increasingly focused on system-level vibration monitoring and fault identification^41^. Advances in modeling nonlinear behaviors and bifurcation in rotating shafts^42^ highlight the sensitivity of the entire assembly to small changes in support conditions. By providing a precise stability map for non-conventional bearing geometries and aggregated nanolubricants, the present work offers the high-fidelity input data required for these comprehensive rotor-system diagnostic frameworks^43^.

### Identification of the research gap and current objectives

Building upon these previous analyses, the current study aims to continue the detailed investigation suggested by Refs.^39^ and^40^ by extending the scope into the dynamic and stability domains. To the best of the authors’ knowledge, no prior literature has considered the stability analysis of hydrodynamic journal bearings featuring variable axial geometrical configurations. This effort seeks to address this critical gap by assessing how changes in lubricant viscosity, driven by nanoparticle concentration, size, and aggregation, influence the stability of bearings with varying axial shapes. TiO₂ is utilized as a representative, data-supported case study to isolate the specific effects of shape and aggregation on stability limitations and dynamic conduct.

The novelty of the present study lies in its integrated dynamic-stability analysis of hydrodynamic journal bearings with variable axial geometrical configurations operating with a TiO2-based nanolubricant while explicitly accounting for nanoparticle aggregation effects on lubricant rheology. Although previous studies have separately examined the stability of conventional journal bearings, the steady-state performance of nanolubricated bearings, and the static behavior of axially modified bearing geometries, the combined influence of axial geometry variation, nanoparticle concentration, and aggregate-induced viscosity modification on the stability limits and nonlinear dynamic response of journal bearings has not been adequately addressed. In this context, the present work extends earlier investigations on plain TiO2-lubricated journal bearings and axially varying geometries by incorporating a modified Krieger-Dougherty viscosity model into a Reynolds-type formulation in curvilinear coordinates and by evaluating the resulting stability thresholds through perturbation analysis and time-domain simulation. This combined framework enables a more realistic assessment of how bearing geometry and aggregation-sensitive nanolubricant behavior interact to govern the transition from stable to unstable operation, thereby providing a more comprehensive basis for the design and performance enhancement of advanced hydrodynamic journal bearings.

## Materials and methods

### Analysis

**Fig. 1:**
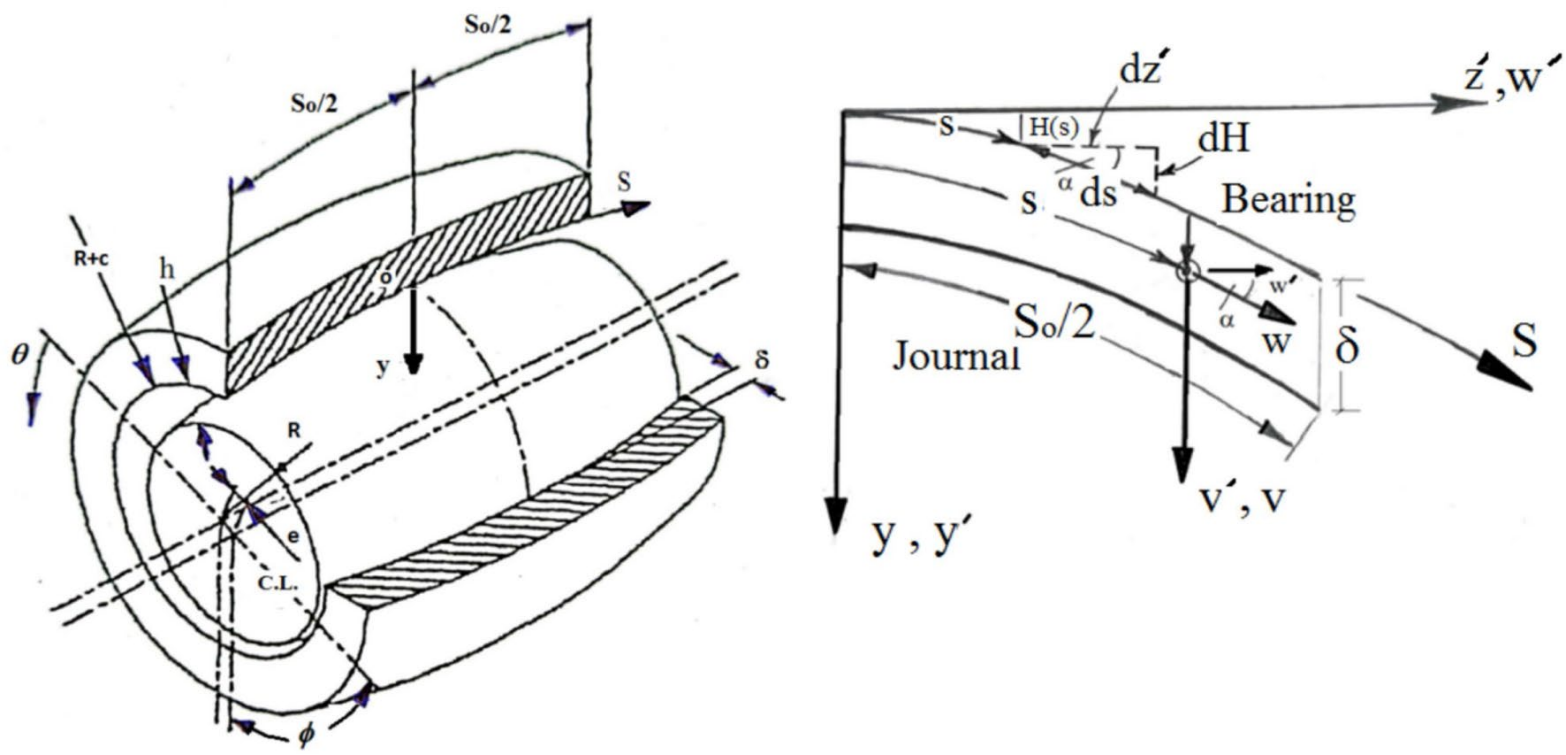
Bearing geometry and curvilinear coordinate system.

Figure 1 depicts the bearing arrangement as well as the curvilinear coordinate system employed in the present research. When the Reynolds number is low, the fluid inertia forces can be ignored in favor of the viscous forces, as is customary in bearing challenges. Using a curvilinear set of coordinates, presented in^40^, a generic form of the Reynolds equation that determines the pressure inside a bearing with changing axial shape may be written as follows,

1 $$\frac{\partial }{{\partial {\mkern 1mu} \theta }}\left( {E{\mkern 1mu} h^{{*^{3} }} \frac{{\partial {\mkern 1mu} \bar{p}}}{{\partial {\mkern 1mu} \theta }}} \right) + \left( {\frac{R}{{S_{o} {\mkern 1mu} E}}} \right)^{2} \frac{\partial }{{\partial {\mkern 1mu} \bar{s}}}\left( {E{\mkern 1mu} h^{{*^{3} }} \frac{{\partial {\mkern 1mu} \bar{p}}}{{\partial {\mkern 1mu} \bar{s}}}} \right) = \frac{6}{E}{\mkern 1mu} \left[ {1 + \frac{{S_{o} }}{R}(\Delta - \bar{H})} \right]{\mkern 1mu} \frac{{\partial {\mkern 1mu} h^{*} }}{{\partial {\mkern 1mu} \theta }} + \frac{{12}}{E}{\mkern 1mu} \frac{{\partial {\mkern 1mu} h^{*} }}{{\partial {\mkern 1mu} \bar{t}}}$$

Where $h^{*}$ is the dimensionless oil film thickness given by $h^{*} = 1 + \varepsilon \,\cos \,(\theta )$, and $H = H(s)$, the function that determines the geometrical configuration of the bearing (see Fig. 1), which must be supplied before to any numerical computations. Also, $E = E(s) = \sqrt {1 - \dot{H}^{2} }$, and the dot represents differentiation with respect to s.

**Fig. 2:**
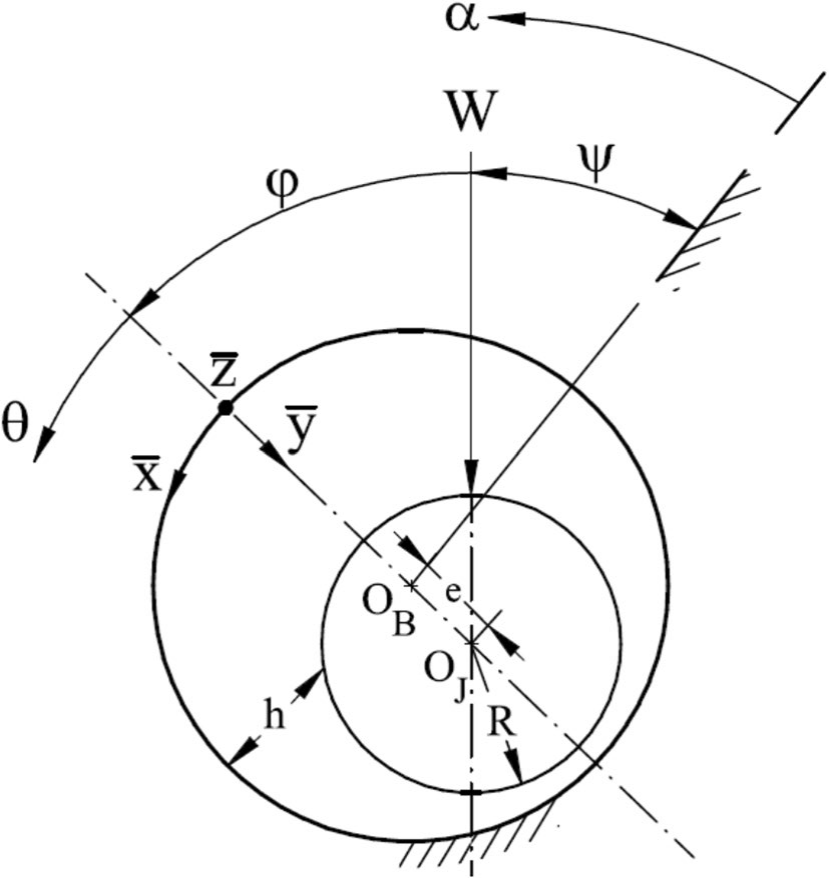
Geometry, coordinate system, and nomenclature of journal bearing.

The angular coordinate $\theta$ is related to a fixed direction through the attitude angle $\phi$ and the instantaneous load direction $\psi$, see Fig. 2. Bearing film thickness $h^{*}$ may be given as follows,

2 $$h^{*} = 1 + \varepsilon {\mkern 1mu} \cos {\mkern 1mu} (\theta ) = 1 + \varepsilon {\mkern 1mu} \cos {\mkern 1mu} (\alpha - (\psi + \phi ))$$

The attitude angle $\phi$ and the instantaneous load angle $\psi$ are used to link the coordinate $\theta$ to a fixed direction *α*, see Fig. 2.

3 $$\frac{{\partial {\mkern 1mu} h^{*} }}{{\partial {\mkern 1mu} \bar{t}}} = \dot{\varepsilon }{\mkern 1mu} \cos {\mkern 1mu} (\theta ) + \varepsilon {\mkern 1mu} \dot{\phi }{\mkern 1mu} \sin {\mkern 1mu} (\theta )$$

Substituting from (3) into (1), the Reynolds equation may be expressed in dimensionless form as,

4 $$\frac{\partial }{{\partial {\mkern 1mu} \theta }}\left( {E{\mkern 1mu} h^{{*^{3} }} \frac{{\partial {\mkern 1mu} \bar{p}}}{{\partial {\mkern 1mu} \theta }}} \right) + \left( {\frac{R}{{S_{o} {\mkern 1mu} E}}} \right)^{2} \frac{\partial }{{\partial {\mkern 1mu} \bar{s}}}\left( {E{\mkern 1mu} h^{{*^{3} }} \frac{{\partial {\mkern 1mu} \bar{p}}}{{\partial {\mkern 1mu} \bar{s}}}} \right) = \frac{6}{E}{\mkern 1mu} \left[ {1 + \left( {\frac{{S_{o} }}{R}} \right)(\Delta - \bar{H})} \right]{\mkern 1mu} \frac{{\partial {\mkern 1mu} h^{*} }}{{\partial {\mkern 1mu} \theta }} + \frac{{12}}{E}{\mkern 1mu} \left( {\dot{\varepsilon }{\mkern 1mu} \cos {\mkern 1mu} (\theta ) + \varepsilon {\mkern 1mu} \dot{\phi }{\mkern 1mu} \sin {\mkern 1mu} (\theta ){\mkern 1mu} } \right)$$

The dot denotes differentiation with respect to the dimensionless time $\overline{t} = \omega \,t$. Substituting $\frac{{\partial \,h^{*} }}{\partial \,\theta } = - \,\varepsilon \,\sin \,(\theta )$ into Eq. (4) yields to,

5 $$\frac{\partial }{{\partial {\mkern 1mu} \theta }}\left( {E{\mkern 1mu} h^{{*^{3} }} \frac{{\partial {\mkern 1mu} \bar{p}}}{{\partial {\mkern 1mu} \theta }}} \right) + \left( {\frac{R}{{S_{o} {\mkern 1mu} E}}} \right)^{2} \frac{\partial }{{\partial {\mkern 1mu} \bar{s}}}\left( {E{\mkern 1mu} h^{{*^{3} }} \frac{{\partial {\mkern 1mu} \bar{p}}}{{\partial {\mkern 1mu} \bar{s}}}} \right) = - \frac{6}{E}{\mkern 1mu} \left[ {\varepsilon {\mkern 1mu} \left( {1 + \left( {\frac{{S_{o} }}{R}} \right)(\Delta - \bar{H}) - 2{\mkern 1mu} \dot{\phi }} \right){\mkern 1mu} \sin {\mkern 1mu} (\theta )} \right]{\mkern 1mu} - 2{\mkern 1mu} \dot{\varepsilon }{\mkern 1mu} \cos {\mkern 1mu} (\theta )$$

To account for the physical occurrence of film rupture in the diverging region of the bearing, the Reynolds boundary condition is used. In this technique, the pressure distribution is solved by converting any expected sub-atmospheric (negative) pressures to ambient (known as the Swift-Stieber condition); both the pressure and its gradient vanish simultaneously. Applying the Swift-Stieber condition, the boundary conditions for the pressure variable $\overline{p}$ may be written as follows,

6 $$\bar{p}{\mkern 1mu} (0,\bar{s}) = 0{\mkern 1mu} {\mkern 1mu} ,{\mkern 1mu} {\mkern 1mu} \bar{p}(\theta _{{eff}} ,\bar{s}) = \left. {\frac{{\partial {\mkern 1mu} \bar{p}}}{{\partial {\mkern 1mu} \theta }}} \right|_{{\theta _{{eff}} }} = 0{\mkern 1mu} {\mkern 1mu} ,{\mkern 1mu} {\mkern 1mu} \bar{p}(\theta ,0) = 0{\mkern 1mu} {\mkern 1mu} {\mkern 1mu} {\mkern 1mu} and{\mkern 1mu} {\mkern 1mu} {\mkern 1mu} {\mkern 1mu} \bar{p}(\theta ,1/2) = 0$$

In dimensionless form the radial and transverse load components are given by,

7 $$\begin{gathered} \bar{W}_{r} = \frac{{W_{r} }}{{\left( {\frac{{\mu _{{bf}} {\mkern 1mu} \omega {\mkern 1mu} R^{3} S_{o} }}{{c^{2} }}} \right)}} = - {\mkern 1mu} 2{\mkern 1mu} {\mkern 1mu} \int\limits_{0}^{{1/2}} {\int\limits_{0}^{{\theta _{{eff}} }} {E{\mkern 1mu} \bar{p}} \left[ {1 + \left( {\frac{{S_{o} }}{R}} \right){\mkern 1mu} \left( {\Delta - \bar{H}} \right)} \right]{\mkern 1mu} {\mkern 1mu} \cos {\mkern 1mu} (\theta ){\mkern 1mu} {\mkern 1mu} d\theta {\mkern 1mu} d{\mkern 1mu} \bar{s}} \hfill \\ \bar{W}_{t} = \frac{{W_{t} }}{{\left( {\frac{{\mu _{{bf}} {\mkern 1mu} \omega {\mkern 1mu} R^{3} S_{o} }}{{c^{2} }}} \right)}} = {\mkern 1mu} {\mkern 1mu} {\mkern 1mu} 2{\mkern 1mu} {\mkern 1mu} \int\limits_{0}^{{1/2}} {\int\limits_{0}^{{\theta _{{eff}} }} {E{\mkern 1mu} \bar{p}} \left[ {1 + \left( {\frac{{S_{o} }}{R}} \right){\mkern 1mu} \left( {\Delta - \bar{H}} \right)} \right]{\mkern 1mu} {\mkern 1mu} {\mkern 1mu} \sin {\mkern 1mu} (\theta ){\mkern 1mu} {\mkern 1mu} d\theta {\mkern 1mu} d{\mkern 1mu} \bar{s}} \hfill \\ \end{gathered}$$

The dimensionless resultant load $\overline{W}$ and the attitude angle $\phi$ may be calculated from,

$$\overline{W} = \left( {\overline{W}_{r}^{2} + \overline{W}_{t}^{2} } \right)^{{{1 \mathord{\left/ {\vphantom {1 2}} \right. \kern-0pt} 2}}} \,\,,\,\,and\,\,\phi = \tan^{ - 1} \left( {\frac{{\overline{W}_{t} }}{{\overline{W}_{r} }}} \right)$$

Integrating the shear stress around the journal surface yields the friction force, which may be expressed in dimensionless form as,

8 $$\begin{gathered} \bar{F}_{f} = \frac{{F_{f} }}{{\left( {\frac{{\mu _{{bf}} {\mkern 1mu} \omega {\mkern 1mu} R^{2} S_{o} }}{c}} \right)}} = {\mkern 1mu} 2{\mkern 1mu} {\mkern 1mu} \int\limits_{0}^{{1/2}} {\int\limits_{0}^{{2{\kern 1pt} \pi }} {E{\mkern 1mu} {\mkern 1mu} \left. {\frac{{\partial {\mkern 1mu} \bar{u}}}{{\partial {\mkern 1mu} \bar{y}}}} \right|_{{\bar{y} = h^{*} }} {\mkern 1mu} } \left[ {1 + \left( {\frac{{S_{o} }}{R}} \right){\mkern 1mu} \left( {\Delta - \bar{H}} \right)} \right]{\mkern 1mu} {\mkern 1mu} {\mkern 1mu} d\theta {\mkern 1mu} d{\mkern 1mu} \bar{s}} \hfill \\ where{\mkern 1mu} {\mkern 1mu} \left. {\frac{{\partial {\mkern 1mu} \bar{u}}}{{\partial {\mkern 1mu} \bar{y}}}} \right|_{{\bar{y} = h^{*} }} = \frac{{E^{2} }}{2}{\mkern 1mu} h^{*} \frac{{\partial {\mkern 1mu} \bar{p}}}{{\partial {\mkern 1mu} \theta }} + \left( {\frac{1}{{h^{*} }}} \right){\mkern 1mu} \left[ {1 + \left( {\frac{{S_{o} }}{R}} \right){\mkern 1mu} \left( {\Delta - \bar{H}} \right)} \right] \hfill \\ \end{gathered}$$

In dimensionless form, the force acting normal to the journal surface is to be calculated from,

9 $$\bar{F}_{n} = \frac{{F_{n} }}{{\left( {\frac{{\mu _{{bf}} {\mkern 1mu} \omega {\mkern 1mu} R^{3} S_{o} }}{{c^{2} }}} \right)}} = \left( {\bar{F}_{{n_{r} }}^{2} + \bar{F}_{{n_{t} }}^{2} } \right)^{{1/2}} {\mkern 1mu}$$

where $$\overline{F}_{{n_{r} }}^{{}} = \frac{{F_{{n_{r} }} }}{{\left( {\frac{{\mu_{bf} \,\omega \,R^{3} S_{o} }}{{c^{2} }}} \right)}} = - \,2\,\,\int\limits_{0}^{{{1 \mathord{\left/ {\vphantom {1 2}} \right. \kern-0pt} 2}}} {} \int\limits_{0}^{{\theta_{eff} }} {\,\overline{p}} \,\cos \,(\theta )\,\left[ {1 + \left( {\frac{{S_{o} }}{R}} \right)\,\left( {\Delta - \overline{H}} \right)} \right]\,\,\,d\theta \,d\,\overline{s}$$.

And $$\overline{F}_{{n_{t} }} = \frac{{F_{{n_{t} }} }}{{\left( {\frac{{\mu_{bf} \,\omega \,R^{3} S_{o} }}{{c^{2} }}} \right)}} = \,\,\,2\,\,\int\limits_{0}^{{{1 \mathord{\left/ {\vphantom {1 2}} \right. \kern-0pt} 2}}} {} \int\limits_{0}^{{\theta_{eff} }} {\,\overline{p}\,\sin \,(\theta )\,} \left[ {1 + \left( {\frac{{S_{o} }}{R}} \right)\,\left( {\Delta - \overline{H}} \right)} \right]\,\,\,d\theta \,d\,\overline{s}$$.

**Fig. 3:**
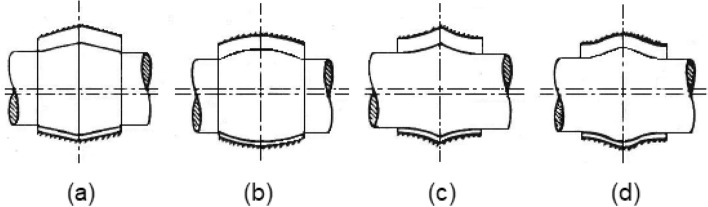
Selected bearing geometrical configurations. (**a**) Wedge; (**b**) Concave; (**c**) Convex; (**d**) Wavy.

The friction parameter (variable) $C_{f} = f\,\left( {{R \mathord{\left/ {\vphantom {R c}} \right. \kern-0pt} c}} \right)$ may be calculated from $f\,\left( {{R \mathord{\left/ {\vphantom {R c}} \right. \kern-0pt} c}} \right) = {{\overline{F}_{f} } \mathord{\left/ {\vphantom {{\overline{F}_{f} } {\overline{F}_{n} }}} \right. \kern-0pt} {\overline{F}_{n} }}$, where $f = {{F_{f} } \mathord{\left/ {\vphantom {{F_{f} } {F_{n} }}} \right. \kern-0pt} {F_{n} }}$. In Fig. 3, four geometrical bearing configurations are considered for comparison with the plain cylindrical bearing. The axial surface profile of the journal is supposed to vary, and four types are considered:

10 $$\begin{gathered} Wedge{\mkern 1mu} {\mkern 1mu} bearing:\bar{H} = 2{\mkern 1mu} {\mkern 1mu} \bar{s}{\mkern 1mu} \Delta \hfill \\ Concave{\mkern 1mu} {\mkern 1mu} axially{\mkern 1mu} {\mkern 1mu} curved{\mkern 1mu} {\mkern 1mu} surfaces:\bar{H} = 4{\mkern 1mu} {\mkern 1mu} \bar{s}^{2} {\mkern 1mu} \Delta \hfill \\ Convex{\mkern 1mu} {\mkern 1mu} axially{\mkern 1mu} {\mkern 1mu} curved{\mkern 1mu} {\mkern 1mu} surfaces:\bar{H} = 4{\mkern 1mu} {\mkern 1mu} \left( {1 - \bar{s}} \right){\mkern 1mu} \bar{s}{\mkern 1mu} \Delta \hfill \\ Wavy{\mkern 1mu} {\mkern 1mu} surfaces:\bar{H} = \frac{1}{2}{\mkern 1mu} \left( {1 - \cos ({\mkern 1mu} 2{\mkern 1mu} \pi {\mkern 1mu} \bar{s}{\mkern 1mu} )} \right){\mkern 1mu} \Delta \hfill \\ \end{gathered}$$

The coordinate $\overline{s}$ can be linked to the coordinate $\overline{z} = {{z^{\prime}} \mathord{\left/ {\vphantom {{z^{\prime}} {S_{o} }}} \right. \kern-0pt} {S_{o} }}$ by,

$$\overline{z} = \int\limits_{0}^{{\overline{s}}} {\sqrt {1 - \left( {{{d\,\overline{H}} \mathord{\left/ {\vphantom {{d\,\overline{H}} {d\,\overline{s}}}} \right. \kern-0pt} {d\,\overline{s}}}} \right)^{2} } \,d\,\overline{s} = \int\limits_{0}^{{\overline{s}}} {E\,d\,\overline{s}} }$$.

The common parameter for the geometries chosen is the greatest variation of the geometry $\overline{H}_{\max } = \Delta$, which should be practically <  < 1. In the present work, computations are conducted for $\Delta = 0.01\,,\,\,0.05\,,\,\,0.1\,,\,\,and\,\,0.15$.

### Viscosity model $\left( {\overline{\mu }} \right)$

While the experimental protocols for TiO₂ nanolubricant preparation and baseline viscosity measurements follow the established methodology of Ref.^22^, recent industrial reviews emphasize that the rheological behavior of nanofluids is fundamentally dictated by the stability and aggregation state of the suspension^44,45^. Modern research confirms that nanoparticles naturally form clusters due to high surface energy, effectively trapping a portion of the base oil within the aggregate structure. This phenomenon increases the effective volume fraction of the solid phase, directly supporting the use of the aggregate packing fraction and the ratio of aggregate-to-primary particle size^46^. Unlike classical models that assume non-interacting spheres, these recent reviews highlight that the aggregation degree is the primary driver of non-Newtonian viscosity shifts and enhanced load-carrying capacity in hydrodynamic bearings^47^. Consequently, the modified Krieger–Dougherty formulation is employed here to provide a physically accurate representation of the lubricant’s resistance to shear in high-speed applications.

The modified Krieger-Dougherty viscosity model may be applied in this study as^15^,

11 $$\bar{\mu } = \frac{{\mu _{{nf}} }}{{\mu _{{bl}} }} = \left( {1 - \frac{{\Phi _{a} }}{{\Phi _{m} }}} \right)^{{{\kern 1pt} - 2.5{\kern 1pt} \Phi _{m} }}$$

With $$\Phi_{a} = \Phi \,\left( {\frac{{a_{a} }}{a}} \right)^{{3 - D^{*} }}$$.

Where,$a_{a} \,\,and\,\,a$ are the radii of aggregates and primary particles. Using the reported values of D^*^ and $\Phi_{m}$, the modified Kriegerr-Dougherty equation may be written as^17^,

12 $$\bar{\mu } = \frac{{\mu _{{nf}} }}{{\mu _{{bl}} }} = \left( {1 - \frac{\Phi }{{\Phi _{m} }}{\mkern 1mu} \left( \beta \right)^{{{\kern 1pt} 1.2}} } \right)^{{{\kern 1pt} - 2.5{\kern 1pt} \Phi _{m} }}$$

**Fig. 4:**
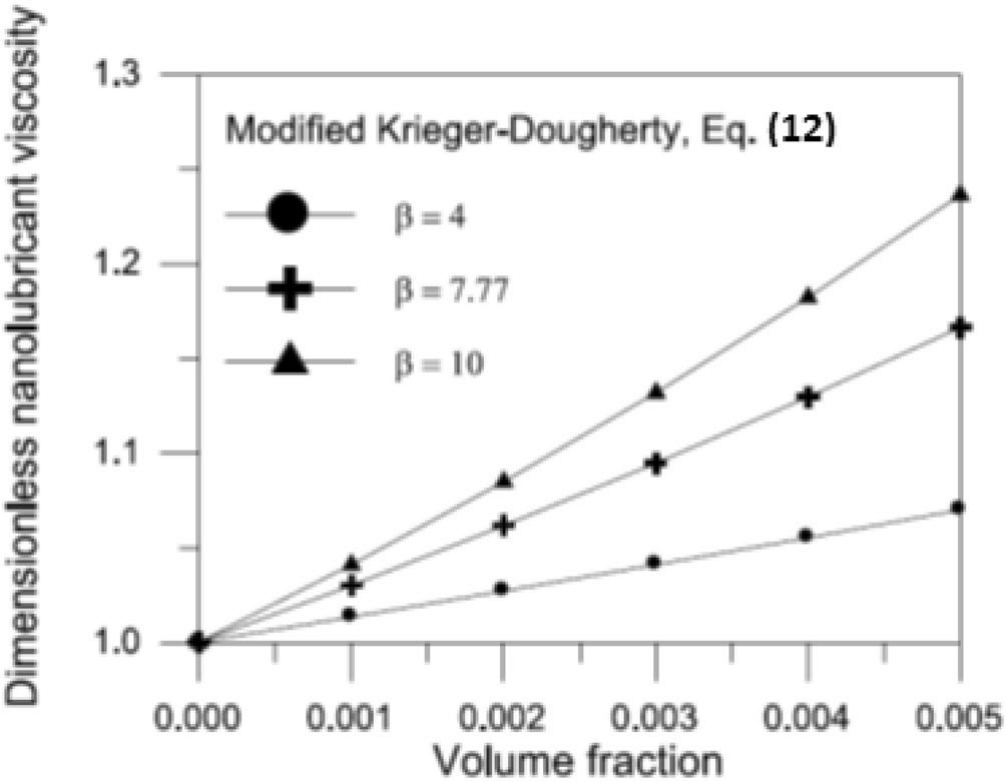
Variation of dimensionless viscosity with TiO_2_ nanoparticle volume fraction for different values of aggregate packing fraction^40^.

Where $\beta = \left( {{{a_{a} } \mathord{\left/ {\vphantom {{a_{a} } a}} \right. \kern-0pt} a}} \right)$ is the aggregate packing fraction, which is determined by the kind and size of the nanoparticles. Binu et al.^22^ measured TiO_2_ nanoparticles (a < 100 nm) distributed in SAE30 engine oil at various volume fractions ($\Phi = 0.0001\,\,to\,\,0.005$). They used DLS analysis to determine the mean aggregate particle size ($a_{a} = 777\,\,nm$) and estimated the aggregate packing fraction ($\beta = 7.77$).This indicates that the TiO_2_ nanoparticle aggregates are approximately 7.77 times the size of the main particle, which is 100 nm. They showed that Eq. (12) agrees fairly well with observed viscosities for various volume fractions. In this study, volume fractions ranged from 0.001 to 0.01 with varying aggregate particle packing fraction values ($\beta = 4\,,\,\,7.77\,\,and\,10$). Figure 4 shows the variation of dimensionless viscosity of TiO_2_ based nano-lubricant *μ* with volume fraction Φ for different values of aggregate particle packing fraction *β*. The findings indicate that *β* has a significant effect on simulating shear viscosities of nanofluids.

### Rotor’s equations of motion

**Fig. 5:**
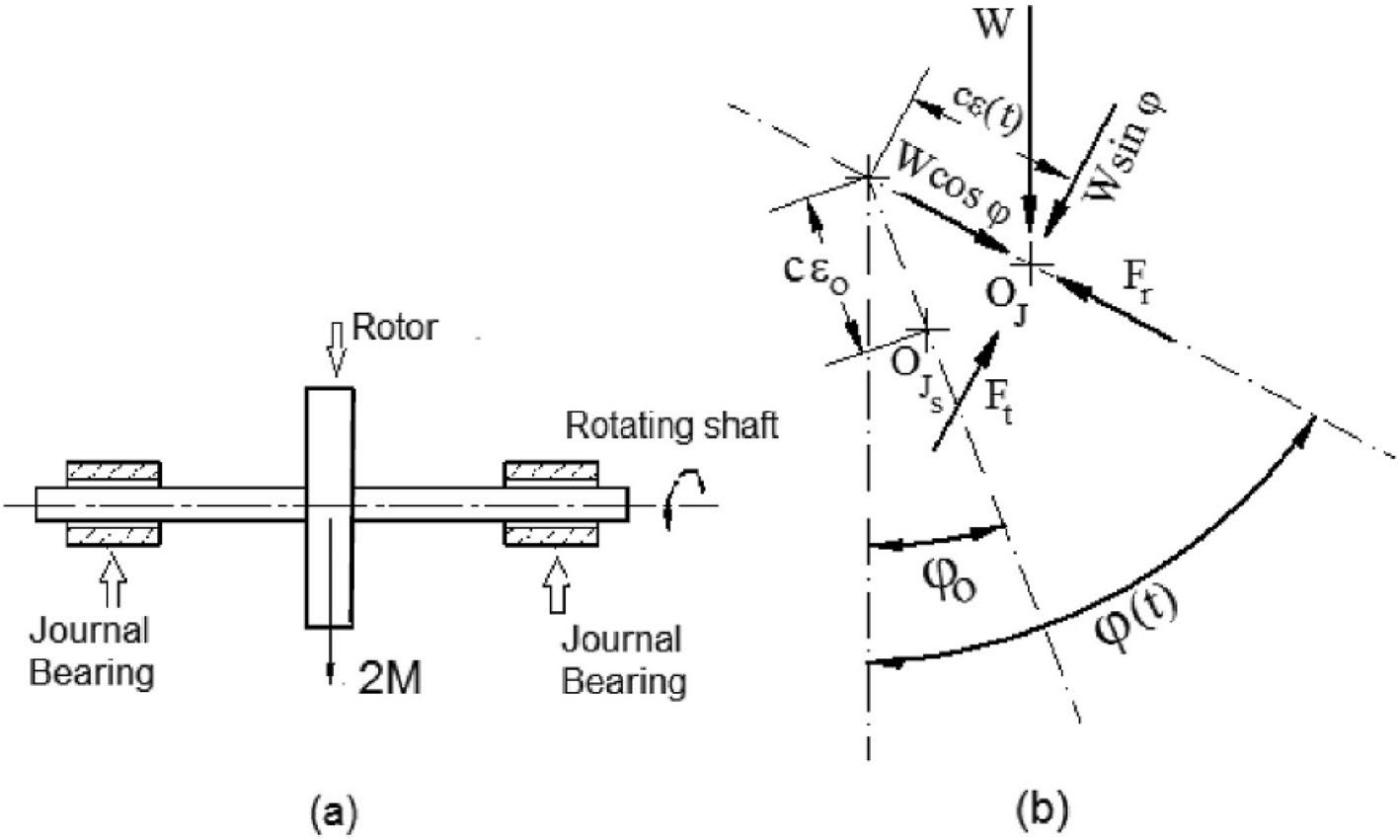
(**a**) Two similar bearings supporting a rigid rotor. (**b**) Forces operating on the bearing journal^39^.

To investigate the stability of journal at equilibrium position, the equations of motion of journal are required. Consider a weightless rigid shaft supporting a disc of mass 2 M at its midpoint. The shaft is supported by identical single pad journal bearings at its ends, as shown in Fig. 5. The equations of motion of the journal are given by:

13 $$\begin{gathered} M{\mkern 1mu} a_{r} = - W_{r} + W{\mkern 1mu} \cos {\mkern 1mu} (\phi ) \hfill \\ M{\mkern 1mu} a_{t} = W_{t} - W{\mkern 1mu} \sin {\mkern 1mu} (\phi ) \hfill \\ \end{gathered}$$

Where $a_{r} \,\,and\,\,a_{t}$ are radial and transverse components of the acceleration,

$$a_{r} = c\,\,\left( {\frac{{d^{2} \varepsilon }}{{d\,t^{2} }} - \varepsilon \,\left( {\frac{d\,\phi }{{d\,t}}} \right)^{2} } \right)\,\,\,\,\,and\,\,\,\,\,\,a_{t} = c\,\,\left( {\varepsilon \,\frac{{d^{2} \phi }}{{d\,t^{2} }} + 2\,\left( {\frac{d\,\varepsilon }{{d\,t}}} \right)\,\left( {\frac{d\,\phi }{{d\,t}}} \right)} \right)$$

In dimensionless form, the equations of motion of the journal are

14 $$\ddot{\varepsilon } - \varepsilon {\mkern 1mu} \dot{\phi }^{2} + \lambda {\mkern 1mu} \left( {\bar{W}_{r} - \bar{W}{\mkern 1mu} \cos {\mkern 1mu} (\phi )} \right) = 0$$

15 $$\varepsilon {\mkern 1mu} \ddot{\phi } + 2{\mkern 1mu} \dot{\varepsilon }{\mkern 1mu} \dot{\phi } - \lambda {\mkern 1mu} \left( {\bar{W}_{t} - \bar{W}{\mkern 1mu} \sin {\mkern 1mu} (\phi )} \right) = 0$$

where $\Lambda$ is the stability number defined as $\lambda = \frac{{\mu_{bf} \,\left( {{R \mathord{\left/ {\vphantom {R c}} \right. \kern-0pt} c}} \right)^{3} S_{o} }}{\omega \,M}$. The dots denote differentiation with respect to $\overline{t}$.

### Perturbation technique

Assuming that the rotor is disturbed and the disturbances are small, the perturbation technique can be used. The variables are assumed to be composed of steady state components and time dependent small oscillations superimposed on them. It can be thus assumed that

16 $$\begin{gathered} \bar{p} = p^{*} (\theta ,\bar{s}) + p^{\prime } (\theta ,\bar{s},\bar{t}) \hfill \\ \varepsilon = \varepsilon _{o} + \varepsilon ^{\prime } (\bar{t}) \hfill \\ h^{*} = h_{o}^{*} + h^{\prime } (\bar{t}) \hfill \\ \phi = \phi _{o} + \phi ^{\prime } (\bar{t}) \hfill \\ \bar{W} = W^{*} + W^{\prime } (\bar{t}) \hfill \\ \end{gathered}$$

Substituting (16) into (5) and neglecting higher order terms yield to the following:

17 $$\begin{gathered} \frac{\partial }{{\partial {\mkern 1mu} \theta }}\left( {E{\mkern 1mu} h_{o}^{{*^{{{\mkern 1mu} {\mkern 1mu} 3}} }} \frac{{\partial {\mkern 1mu} p^{*} }}{{\partial {\mkern 1mu} \theta }} + E{\mkern 1mu} h_{o}^{{*^{{{\mkern 1mu} {\mkern 1mu} 3}} }} \frac{{\partial {\mkern 1mu} p^{\prime } }}{{\partial {\mkern 1mu} \theta }} + 3{\mkern 1mu} E{\mkern 1mu} h_{o}^{{*^{{{\mkern 1mu} {\mkern 1mu} 2}} }} h^{\prime } \frac{{\partial {\mkern 1mu} p^{*} }}{{\partial {\mkern 1mu} \theta }}} \right) \hfill \\ {\mkern 1mu} {\mkern 1mu} {\mkern 1mu} {\mkern 1mu} {\mkern 1mu} {\mkern 1mu} {\mkern 1mu} {\mkern 1mu} {\mkern 1mu} {\mkern 1mu} {\mkern 1mu} {\mkern 1mu} {\mkern 1mu} {\mkern 1mu} {\mkern 1mu} {\mkern 1mu} {\mkern 1mu} {\mkern 1mu} {\mkern 1mu} {\mkern 1mu} {\mkern 1mu} {\mkern 1mu} {\mkern 1mu} {\mkern 1mu} {\mkern 1mu} {\mkern 1mu} {\mkern 1mu} + \left( {\frac{R}{{S_{o} {\mkern 1mu} E}}} \right)^{2} \frac{\partial }{{\partial {\mkern 1mu} \bar{s}}}\left( {E{\mkern 1mu} h_{o}^{{*^{{{\mkern 1mu} {\mkern 1mu} 3}} }} \frac{{\partial {\mkern 1mu} p^{*} }}{{\partial {\mkern 1mu} \bar{s}}} + E{\mkern 1mu} h_{o}^{{*^{{{\mkern 1mu} {\mkern 1mu} 3}} }} \frac{{\partial {\mkern 1mu} p^{\prime } }}{{\partial {\mkern 1mu} \bar{s}}} + 3{\mkern 1mu} E{\mkern 1mu} h_{o}^{{*^{2} }} h^{\prime } \frac{{\partial {\mkern 1mu} p^{*} }}{{\partial {\mkern 1mu} \bar{s}}}} \right) \hfill \\ {\mkern 1mu} {\mkern 1mu} {\mkern 1mu} {\mkern 1mu} {\mkern 1mu} {\mkern 1mu} {\mkern 1mu} {\mkern 1mu} {\mkern 1mu} {\mkern 1mu} {\mkern 1mu} {\mkern 1mu} {\mkern 1mu} {\mkern 1mu} {\mkern 1mu} {\mkern 1mu} {\mkern 1mu} {\mkern 1mu} {\mkern 1mu} {\mkern 1mu} {\mkern 1mu} {\mkern 1mu} {\mkern 1mu} {\mkern 1mu} {\mkern 1mu} {\mkern 1mu} {\mkern 1mu} {\mkern 1mu} {\mkern 1mu} = {\mkern 1mu} {\mkern 1mu} - \frac{6}{E}\varepsilon _{o} {\mkern 1mu} \left( {1 + \left( {\frac{{S_{o} }}{R}} \right)(\bar{\Delta } - \bar{H})} \right){\mkern 1mu} \sin {\mkern 1mu} (\theta ){\mkern 1mu} - \frac{6}{E}\varepsilon ^{\prime } {\mkern 1mu} \left( {1 + \left( {\frac{{S_{o} }}{R}} \right)(\Delta - \bar{H})} \right){\mkern 1mu} \sin {\mkern 1mu} (\theta ){\mkern 1mu} \hfill \\ {\mkern 1mu} {\mkern 1mu} {\mkern 1mu} {\mkern 1mu} {\mkern 1mu} {\mkern 1mu} {\mkern 1mu} {\mkern 1mu} {\mkern 1mu} {\mkern 1mu} {\mkern 1mu} {\mkern 1mu} {\mkern 1mu} {\mkern 1mu} {\mkern 1mu} {\mkern 1mu} {\mkern 1mu} {\mkern 1mu} {\mkern 1mu} {\mkern 1mu} {\mkern 1mu} {\mkern 1mu} {\mkern 1mu} {\mkern 1mu} {\mkern 1mu} {\mkern 1mu} {\mkern 1mu} {\mkern 1mu} {\mkern 1mu} {\mkern 1mu} + \frac{{12}}{E}{\mkern 1mu} \left( {\varepsilon _{o} {\mkern 1mu} \dot{\phi }^{\prime } + {\mkern 1mu} \dot{\varepsilon }^{\prime } {\mkern 1mu} \cos {\mkern 1mu} (\theta )} \right) \hfill \\ \end{gathered}$$

Separating the steady state components and time dependent small oscillations, the governing equations are

18 $$\frac{\partial }{{\partial {\mkern 1mu} \theta }}\left( {E{\mkern 1mu} h_{o}^{{*{\kern 1pt} ^{3} }} \frac{{\partial {\mkern 1mu} p^{*} }}{{\partial {\mkern 1mu} \theta }}} \right) + \left( {\frac{R}{{S_{o} {\mkern 1mu} E}}} \right)^{2} \frac{\partial }{{\partial {\mkern 1mu} \bar{s}}}\left( {E{\mkern 1mu} h_{o}^{{*{\kern 1pt} ^{3} }} \frac{{\partial {\mkern 1mu} p^{*} }}{{\partial {\mkern 1mu} \bar{s}}}} \right) = {\mkern 1mu} {\mkern 1mu} - \frac{6}{E}\varepsilon _{o} {\mkern 1mu} \left( {1 + \left( {\frac{{S_{o} }}{R}} \right)(\Delta - \bar{H})} \right){\mkern 1mu} \sin {\mkern 1mu} (\theta )$$

And,

19 $$\begin{gathered} \frac{\partial }{{\partial {\mkern 1mu} \theta }}\left( {E{\mkern 1mu} h_{o}^{{*^{{{\mkern 1mu} {\mkern 1mu} 3}} }} \frac{{\partial {\mkern 1mu} p^{\prime } }}{{\partial {\mkern 1mu} \theta }}} \right){\mkern 1mu} + \left( {\frac{R}{{S_{o} {\mkern 1mu} E}}} \right)^{2} \frac{\partial }{{\partial {\mkern 1mu} \bar{s}}}\left( {E{\mkern 1mu} h_{o}^{{*^{{{\mkern 1mu} {\mkern 1mu} 3}} }} \frac{{\partial {\mkern 1mu} p^{\prime } }}{{\partial {\mkern 1mu} \bar{s}}}} \right) \hfill \\ {\mkern 1mu} {\mkern 1mu} {\mkern 1mu} {\mkern 1mu} {\mkern 1mu} {\mkern 1mu} {\mkern 1mu} {\mkern 1mu} {\mkern 1mu} {\mkern 1mu} {\mkern 1mu} {\mkern 1mu} {\mkern 1mu} {\mkern 1mu} {\mkern 1mu} {\mkern 1mu} {\mkern 1mu} {\mkern 1mu} {\mkern 1mu} {\mkern 1mu} {\mkern 1mu} = - \frac{\partial }{{\partial {\mkern 1mu} \theta }}\left( {3{\mkern 1mu} E{\mkern 1mu} h_{o}^{{*^{{{\mkern 1mu} {\mkern 1mu} 2}} }} h^{\prime } \frac{{\partial {\mkern 1mu} p^{*} }}{{\partial {\mkern 1mu} \theta }}} \right) - \left( {\frac{R}{{S_{o} {\mkern 1mu} E}}} \right)^{2} \frac{\partial }{{\partial {\mkern 1mu} \bar{s}}}\left( {3{\mkern 1mu} E{\mkern 1mu} h_{o}^{{*^{{{\mkern 1mu} {\mkern 1mu} 2}} }} h^{\prime } \frac{{\partial {\mkern 1mu} p^{*} }}{{\partial {\mkern 1mu} \bar{s}}}} \right) \hfill \\ {\mkern 1mu} {\mkern 1mu} {\mkern 1mu} {\mkern 1mu} {\mkern 1mu} {\mkern 1mu} {\mkern 1mu} {\mkern 1mu} {\mkern 1mu} {\mkern 1mu} {\mkern 1mu} {\mkern 1mu} {\mkern 1mu} {\mkern 1mu} {\mkern 1mu} {\mkern 1mu} {\mkern 1mu} {\mkern 1mu} {\mkern 1mu} {\mkern 1mu} {\mkern 1mu} {\mkern 1mu} {\mkern 1mu} {\mkern 1mu} {\mkern 1mu} {\mkern 1mu} {\mkern 1mu} {\mkern 1mu} {\mkern 1mu} {\mkern 1mu} {\mkern 1mu} {\mkern 1mu} {\mkern 1mu} {\mkern 1mu} {\mkern 1mu} {\mkern 1mu} {\mkern 1mu} {\mkern 1mu} - \frac{6}{E}\varepsilon ^{\prime } {\mkern 1mu} \left( {1 + \left( {\frac{{S_{o} }}{R}} \right)(\Delta - \bar{H})} \right){\mkern 1mu} \sin {\mkern 1mu} (\theta ){\mkern 1mu} + \frac{{12}}{E}{\mkern 1mu} \left( {\varepsilon _{o} {\mkern 1mu} \dot{\phi }^{\prime } + {\mkern 1mu} \dot{\varepsilon }^{\prime } {\mkern 1mu} \cos {\mkern 1mu} (\theta )} \right) \hfill \\ \end{gathered}$$

Equation (18) is the steady state equilibrium equation and Eq. (19) is a linear perturbation equation since higher order terms are neglected.

Since $h^{*} = 1 + (\varepsilon_{o} + \varepsilon^{\prime})\,\cos \,(\theta ) = (1 + \varepsilon_{o} \cos \,(\theta )) + \varepsilon^{\prime}\,\cos \,(\theta ) = h_{o}^{*} + h^{\prime}$, then $h^{\prime} = \varepsilon^{\prime}\,\cos \,(\theta )$. Equation (19) may be written in a concise form as follows,

20 $$\begin{gathered} \frac{\partial }{{\partial {\mkern 1mu} \theta }}\left( {E{\mkern 1mu} h_{o}^{{*^{{{\mkern 1mu} {\mkern 1mu} 3}} }} \frac{{\partial {\mkern 1mu} p^{\prime } }}{{\partial {\mkern 1mu} \theta }}} \right){\mkern 1mu} + \left( {\frac{R}{{S_{o} {\mkern 1mu} E}}} \right)^{2} \frac{\partial }{{\partial {\mkern 1mu} \bar{s}}}\left( {E{\mkern 1mu} h_{o}^{{*^{{{\mkern 1mu} {\mkern 1mu} 3}} }} \frac{{\partial {\mkern 1mu} p^{\prime } }}{{\partial {\mkern 1mu} \bar{s}}}} \right){\mkern 1mu} \hfill \\ {\text{ }}{\mkern 1mu} {\mkern 1mu} {\mkern 1mu} {\mkern 1mu} {\mkern 1mu} {\mkern 1mu} {\mkern 1mu} {\mkern 1mu} {\mkern 1mu} {\mkern 1mu} {\mkern 1mu} {\mkern 1mu} {\mkern 1mu} {\mkern 1mu} {\mkern 1mu} {\mkern 1mu} {\mkern 1mu} {\mkern 1mu} {\mkern 1mu} {\mkern 1mu} = - \frac{6}{E}\left\{ {\varepsilon ^{\prime } {\mkern 1mu} \left[ {\left( {1 + \left( {\frac{{S_{o} }}{R}} \right)(\Delta - \bar{H})} \right){\mkern 1mu} \sin {\mkern 1mu} (\theta ){\mkern 1mu} + P(\theta ,\bar{s})} \right]{\mkern 1mu} - 2{\mkern 1mu} \varepsilon _{o} {\mkern 1mu} \dot{\phi }^{\prime } - 2{\mkern 1mu} \dot{\varepsilon }^{\prime } {\mkern 1mu} \cos {\mkern 1mu} (\theta )} \right\} \hfill \\ \end{gathered}$$

where, $$P(\theta ,\overline{s}) = \frac{E}{2}\,\left\{ {\frac{\partial }{\partial \,\theta }\left( {\,E\,h_{o}^{{*^{\,2} }} \cos (\theta )\frac{{\partial \,p^{*} }}{\partial \,\theta }} \right)} \right\} + \left( {\frac{R}{{S_{o} \,E}}} \right)^{2} \frac{\partial }{{\partial \,\overline{s}}}\left( {\,E\,h_{o}^{{*^{\,2} }} \cos (\theta )\frac{{\partial \,p^{*} }}{{\partial \,\overline{s}}}} \right)$$.

The boundary conditions for Eqs. (18) and (20) are

21 $$\begin{gathered} p^{*} {\mkern 1mu} (0,\bar{s}) = 0{\mkern 1mu} {\mkern 1mu} ,{\mkern 1mu} {\mkern 1mu} p^{*} (\theta _{{eff}} ,\bar{s}) = \left. {\frac{{\partial {\mkern 1mu} p^{*} }}{{\partial {\mkern 1mu} \theta }}} \right|_{{\theta _{{eff}} }} = 0{\mkern 1mu} {\mkern 1mu} ,{\mkern 1mu} {\mkern 1mu} p^{*} (\theta ,1/2) = 0{\mkern 1mu} ,{\mkern 1mu} {\mkern 1mu} {\mkern 1mu} \frac{{\partial {\mkern 1mu} p^{*} (\theta ,0)}}{{\partial {\mkern 1mu} \bar{s}}}{\mkern 1mu} = 0 \hfill \\ p^{\prime } {\mkern 1mu} (0,\bar{s}) = 0{\mkern 1mu} {\mkern 1mu} ,{\mkern 1mu} {\mkern 1mu} p^{\prime } (\theta _{{eff}} ,\bar{s}) = \left. {\frac{{\partial {\mkern 1mu} p^{\prime } }}{{\partial {\mkern 1mu} \theta }}} \right|_{{\theta _{{eff}} }} = 0{\mkern 1mu} {\mkern 1mu} ,{\mkern 1mu} {\mkern 1mu} p^{\prime } (\theta ,1/2) = 0{\mkern 1mu} ,{\mkern 1mu} {\mkern 1mu} {\mkern 1mu} \frac{{\partial {\mkern 1mu} p^{\prime } (\theta ,0)}}{{\partial {\mkern 1mu} \bar{s}}}{\mkern 1mu} = 0{\mkern 1mu} \hfill \\ \end{gathered}$$

The perturbation load components are

22 $$W_{r}^{\prime } = - {\mkern 1mu} 2{\mkern 1mu} {\mkern 1mu} \int\limits_{0}^{{1/2}} {\int\limits_{0}^{{\theta _{{eff}} }} {E{\mkern 1mu} p^{\prime } {\mkern 1mu} {\mkern 1mu} } \left[ {1 + \left( {\frac{{S_{o} }}{R}} \right){\mkern 1mu} \left( {\Delta - \bar{H}} \right)} \right]{\mkern 1mu} {\mkern 1mu} \cos {\mkern 1mu} (\theta ){\mkern 1mu} {\mkern 1mu} d\theta {\mkern 1mu} d{\mkern 1mu} \bar{s}}$$

And,

23 $$W_{t}^{\prime } = {\mkern 1mu} {\mkern 1mu} {\mkern 1mu} 2{\mkern 1mu} {\mkern 1mu} \int\limits_{0}^{{1/2}} {\int\limits_{0}^{{\theta _{{eff}} }} {E{\mkern 1mu} p^{\prime } {\mkern 1mu} {\mkern 1mu} } \left[ {1 + \left( {\frac{{S_{o} }}{R}} \right){\mkern 1mu} \left( {\Delta - \bar{H}} \right)} \right]{\mkern 1mu} {\mkern 1mu} {\mkern 1mu} \sin {\mkern 1mu} (\theta ){\mkern 1mu} {\mkern 1mu} d\theta {\mkern 1mu} d{\mkern 1mu} \bar{s}}$$

Assuming $W^{\prime} = 0$, the perturbation equations governing the motion of the journal are

24 $$\ddot{\varepsilon }^{\prime } - (\varepsilon _{o} + \varepsilon ^{\prime } ){\mkern 1mu} \dot{\phi }^{{{\prime } ^{2} }} + \lambda {\mkern 1mu} \left( {W_{r}^{\prime } + W^{*} {\mkern 1mu} \phi ^{\prime } {\mkern 1mu} \sin {\mkern 1mu} {\mkern 1mu} (\phi _{o} )} \right) = 0$$

And,

25 $$(\varepsilon _{o} + \varepsilon ^{\prime } ){\mkern 1mu} \ddot{\phi }^{\prime } + 2{\mkern 1mu} \dot{\varepsilon }^{\prime } {\mkern 1mu} \dot{\phi }^{\prime } - \lambda {\mkern 1mu} \left( {W_{t}^{\prime } - W^{*} {\mkern 1mu} \phi ^{\prime } {\mkern 1mu} \cos {\mkern 1mu} {\mkern 1mu} (\phi _{o} )} \right)$$

The initial conditions for Eqs. (24) and (25) are

$$\varepsilon^{\prime}(0) = \varepsilon_{i} \,\,and\,\,\dot{\varepsilon }^{\prime}(0) = \phi^{\prime}(0) = \dot{\phi }^{\prime}(0) = 0$$

### Solution methodology

The finite difference method was used to numerically solve the governing Eqs. (18) and (20) and the corresponding boundary conditions (21). All negative pressure values are set to zero during the solution process^21^, and the resulting sets of simultaneous equations are solved using successive over relaxation technique. The iteration process keeps going until convergence is reached with a relative tolerance of 0.01. A FORTRAN code in the discretized Reynolds equations uses the modified Krieger-Dougherty model, Eq. (12), to determine the steady state and time dependent pressure distributions in bearing lubricant film. The bearing characteristics of various TiO_2_ nanoparticle volume fractions have been evaluated at different values of nanoparticle aggregate size. The coupled nonlinear perturbation dynamic Eqs. (24) and (25) with their initial conditions are solved using the Runge–Kutta stiff approach at a dimensionless time step of 0.01.

## Results and discussion

### Steady state characteristics

**Fig. 6:**
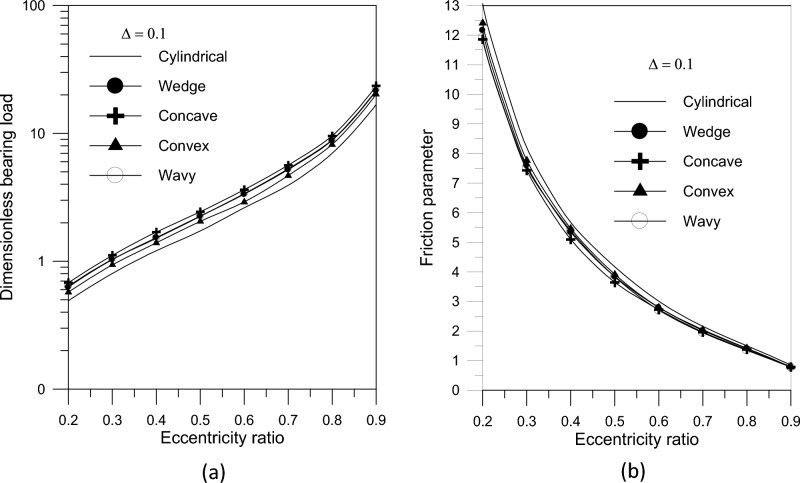
Bearing characteristics (bearing load and friction) for different geometries with ${{\Phi = 0\,\,and\,\,S_{o} } \mathord{\left/ {\vphantom {{\Phi = 0\,\,and\,\,S_{o} } {D = 1}}} \right. \kern-0pt} {D = 1}}$^40^.

Awad et al.^40^ studied into how the behavior of journal bearings in steady state was greatly affected by the usage of journal bearings with an axial variation in geometrical shape and the addition of nanoparticles as lubricant additives. Four different forms were used to create the bearing: wavy, concave, convex, and wedge. The modified Krieger-Dougherty model, which is based on the experimental work of Binu et al.^22^, is used to determine the viscosity of the nanolubricant. The nanolubricant was formulated using SAE30 engine oil and TiO_2_ nanoparticles (scale 100 nm); the volume fractions for various nanoparticle aggregate sizes vary from 0.001 to 0.01. A concave surface generates higher pressure levels inside the fluid film, according to their analysis of the bearing’s mid-plane pressure distribution. The pressure distribution values in the bearing oil film increase with the maximum geometric variation. Additionally, the pressure values in the bearing fluid film increase with the eccentricity ratio. It was noteworthy that every geometry proved to be able to support a greater load than a typical cylindrical bearing. This held true for all ratios of eccentricity. The load carrying capacity rises with the eccentricity ratio for all geometries. The concave shape had the maximum load carrying capacity at any eccentricity ratio when the geometries were examined, Fig. 6a. They calculated the friction parameter and discovered that it decreased for all of the geometries considered when compared to the simple cylindrical journal bearing. The superiority of concave geometry over other geometries was evident, Fig. 6b. The findings demonstrated that increasing the geometric variation increases the bearing load capacity of concave axially curved surfaces.

Furthermore, the largest change in concave surface form results in a significant reduction in friction parameter at low eccentricity ratios. Their studies revealed that the presence of TiO_2_ nanoparticle lubricant additives improves the bearing load carrying capacity. The increase in bearing load was shown to be more evident at higher TiO_2_ nanoparticle volume fraction levels. For a given volume fraction, the results demonstrated that raising the aggregate packing fraction expands bearing load capacity but has no apparent effect on friction parameter. In comparison to simple cylindrical bearings, the results for the bearing geometries under consideration showed that slight changes in the axial shape of the bearing increased load carrying capacity while decreasing the friction parameter (variable). Concave geometry outperforms other geometries. Furthermore, utilizing TiO_2_ nanoparticles as a lubricant additive improves bearing load capacity without affecting friction parameters. The choice of a concave bearing surface shape with a dimensionless maximum axial variation $\Delta = 0.1$ and a nanofluid lubricant with a volume fraction $\Delta = 0.1$ and an aggregate packing fraction $\beta = 7.77$ causes a relative change in bearing characteristics, as shown in Table 1.

**Table 1:** Relative difference in bearing characteristics for concave bearing surface shape with and without lubricant additives^40^.

$\varepsilon$	Surface geometry		Nanofluid lubricant		Combination of the two	
	$\% \,W_{\,g}$	$\% \,C_{f\,g}$	$\% \,W_{\,n}$	$\% \,C_{f\,n}$	$\% \,W_{\,gn}$	$\% \,C_{f\,g\,n}$
0.2	38.412	− 9.253	16.611	− 0.003	61.404	− 9.255
0.3	38.489	− 9.303	16.611	− 0.00376	61.494	− 9.307
0.4	39.647	− 10.056	16.611	− 0.0042	62.844	− 10.06
0.5	41.553	− 11.268	16.611	− 0.0039	65.067	− 11.271
0.6	38.403	− 9.247	16.611	− 0.00294	61.394	− 9.25
0.7	43.767	− 12.635	16.611	− 0.00138	67.649	− 12.636
0.8	37.2	− 8.448	16.611	− 0.00225	59.991	− 8.45
0.9	37.191	− 8.453	16.611	− 0.0256	59.98	− 8.519
			TiO_2_ lubricant additive: ϕ = 0.005 and β = 7.77			

When compared to a plain cylindrical bearing, the results obtained for the bearing geometries under consideration demonstrate that modifications in the axial shape of the bearing enhance the load carrying capacity and lower the friction parameter (variable). This is especially noticeable for bearings that are comparatively lengthy. Increasing the nanoparticle volume fraction leads to higher bearing load capacity and lower friction. The bearing load capacity increases as the aggregate packing fraction increases, with little impact on the friction characteristic.

### Examination of stability for various geometries

**Fig. 7:**
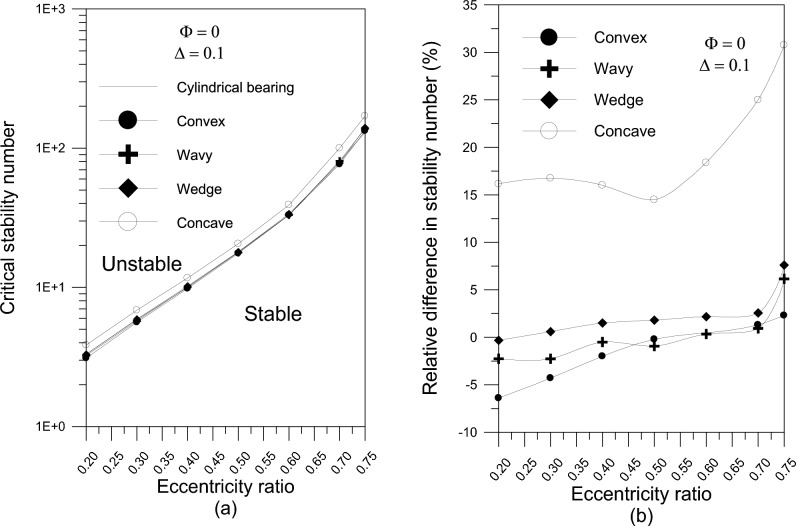
(**a**) The stability number versus eccentricity ratio for different bearing geometries. (**b**) The relative difference in stability number versus eccentricity ratio.

This part examines the effect of various axial geometrical configurations on the stability of fluid film bearings of finite width in oil-lubricated journal bearings without using nanoparticle additives. Figure 7a illustrates the stability map for a rigid rotor with different bearing surface forms. For the examined bearing geometries compared to the typical critical stability number for cylindrical bearing for different values of eccentricity ratio, Fig. 7b shows the relative difference in critical stability number, $\% \,\lambda_{cr} = ((\lambda_{cr} - \lambda_{o} )/\lambda_{o} ) \times 100$, where $\lambda_{o}$ is the critical value of the stability number in the case of cylindrical bearing. Concave and wedge geometries outperform other shapes, however the results reveal that the concave geometry is always advised to get the highest critical stability number for a given eccentricity ratio.

### Dynamic behavior and stability limits

**Fig. 8:**
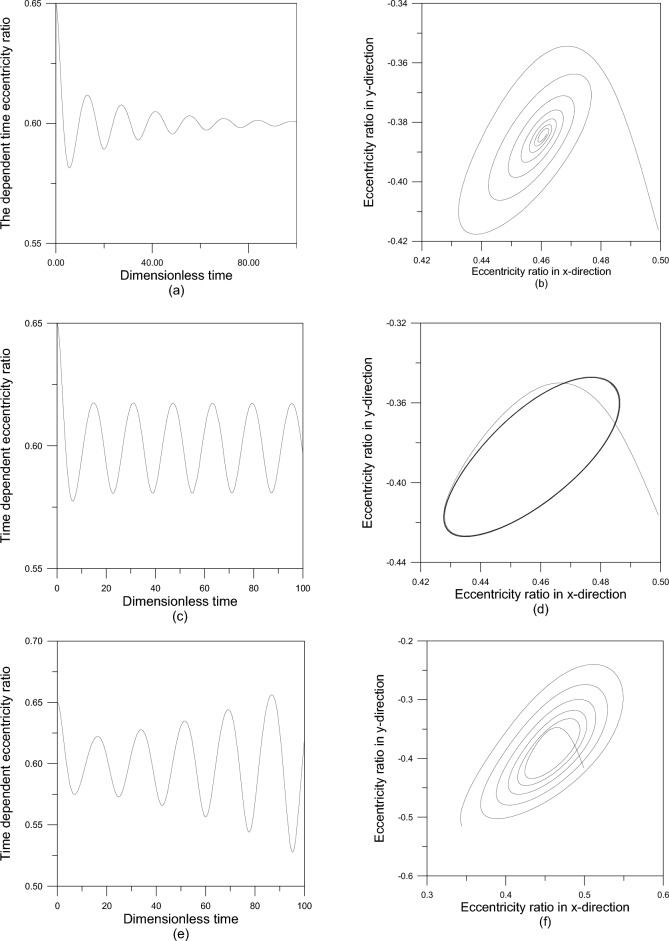
Vibration behavior and rotor trajectory at $\varepsilon_{o} = 0.6\,,\,\,\varepsilon^{\prime}(0) = 0.05$ using modified Krierger-Dougherty viscosity model with $\Phi = 0.005\,\,and\,\,\beta = 7.77$: (**a** & **b**) stable oscillations ($\lambda = 36$); (**c** & **d**) critical case ($\lambda_{cr} = 44.125)$ and (**e** & **f**) unstable oscillations ($\lambda = 56)$.

The current study examines the effect of various axial geometrical configurations on the stability of fluid film bearings of finite width in oil-lubricated journal bearings using titanium dioxide nanoparticles as lubricant additives. The journal’s vibration behavior is numerically simulated to analyze the dynamics of it and to determine the bearing’s stability limits. The trajectory or orbit represents the motion of the journal center. When stable oscillations are present, the shaft (journal) center converges to the equilibrium position, but unstable oscillations cause the shaft center to stay mobile for an infinite amount of time, resulting in bearing failure. If the journal center moves and follows a closed orbit around its equilibrium location, this is an example of instability or critical stability. The rotor’s oscillations in the x and y directions, as well as the time-dependent overall eccentricity ratio $\varepsilon (t)$, are determined by the stability number $\lambda$. In this regard, Fig. 8 depicts the vibration behavior and rotor center trajectory (orbit) at $\varepsilon_{o} = 0.6$ and $\varepsilon^{\prime}(0) = 0.05$, with $\Phi = 0.003$ and $\beta = 7.77$.

As seen in Fig. 8a and b, at $\lambda = 30 < \lambda_{cr}$, there are steady oscillations and the rotor center goes progressively closer to the equilibrium position. Bearing failure occurs when the rotor center remains mobile indefinitely due to unstable oscillations (Fig. 8e and f), where $\lambda = 45 > \lambda_{cr}$. The response of the rotor exhibits sinusoidal sustained oscillations for $\lambda = \lambda_{cr} = 40.81$, and the rotor center moves in a closed orbit around its equilibrium location (Fig. 8c and d). In the critical or stationary case, the stability number is referred to as the critical stability number $\lambda_{cr}$, and the distinction between stable and unstable conditions is established.

**Fig. 9:**
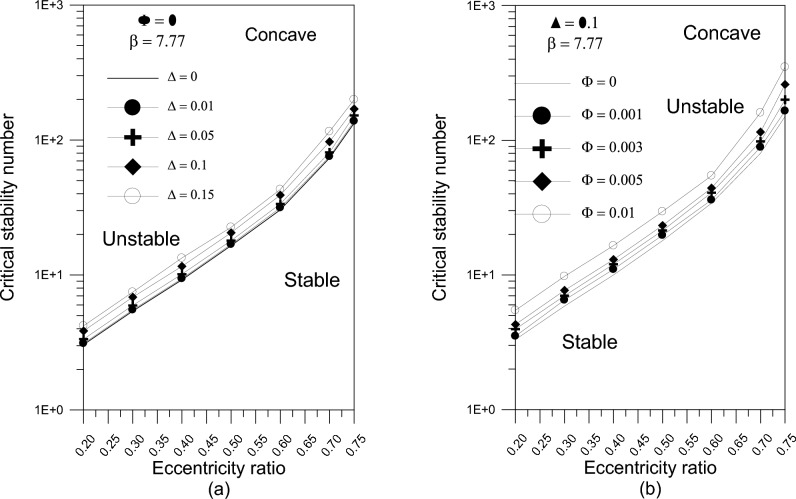
(**a**) Effect of maximum axial variation on critical stability number of the bearing. (**b**) Effect of nanoparticle volume fraction on bearing critical stability number.

The results in Fig. 9a show that increasing the greatest variation of geometry $\Delta$ raises the value of the stability number for a concave axially curved surface bearing. As shown in Fig. 9b, the influence of different TiO_2_ volume fractions on the bearing critical stability number for concave surface bearings is investigated and predicted. The results reveal that the use of TiO_2_ nanoparticle lubricant additives raises the critical stability number, which is more apparent at higher TiO_2_ nanoparticle volume percent values.

**Fig. 10:**
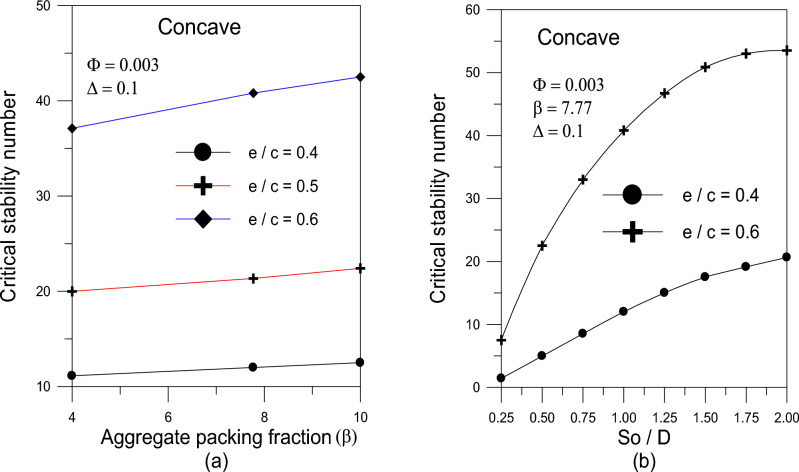
(**a**) Effect of packing volume fraction on critical stability number. (**b**) Effect of ${{S_{o} } \mathord{\left/ {\vphantom {{S_{o} } D}} \right. \kern-0pt} D}$ on critical stability number.

The influence of the aggregate packing percent was studied at $\beta$ values of 4, 7.77, and 10. The results reveal that increasing the value of $\beta$ leads to a rise in the critical stability number $\lambda_{cr}$, as shown in Fig. 10a. Figure 10a depicts the effect of the aggregate packing fraction ($\beta$) on the critical stability number ($\lambda_{cr}$). While it is clear that a higher packing fraction raises the stability threshold for all eccentricity ratios, the data also show a substantial interaction effect with the bearing’s axial shape. The concave form is far more sensitive to changes in ($\beta$) than the convex or wavy surfaces. This shows that the concave geometry’s higher center film thickness acts as a ‘buffer’, allowing the increased effective viscosity resulting from high aggregate packing to be translated into damping energy more efficiently while avoiding premature film rupture. Additionally, the interaction with nanoparticle volume fraction ($\Phi$) is non-linear; at low volume concentrations, the geometric profile has a greater influence than the highest value of volume fraction concentration. However, the aggregation state takes over as the primary driver of stability when the volume percentage rises, hence reducing the performance difference between the various axial forms. This suggests that maintaining a stable operating margin for severely loaded bearings depends more on controlling nanoparticle stability and cluster size than it does on physically modifying the axial profile. The effect of bearing length on the value of the critical stability number is illustrated in Fig. 10b. As the bearing length increases the critical stability number increases.

## Conclusions

The current study looks at how adopting an axial geometrical design for hydrodynamic bearings lubricated with nanolubricant comprising titanium dioxide nanoparticles as lubricant additives affects the bearing’s dynamic performance and stability limits. A curvilinear coordinate system is used to generate the Reynolds-like equation that determines pressure within the bearings. The governing equations for dynamic settings are derived using a perturbation technique. This research demonstrates that the dynamic stability of high-speed rotors can be significantly enhanced through the strategic combination of axial bearing profiling and nanofluid additives. The primary finding identifies the concave axial configuration as the optimal design choice, consistently providing a superior stability threshold compared to conical, convex, or wavy geometries.

Furthermore, by utilizing a modified Krieger-Dougherty model, this study proves that nanoparticle aggregation is a dominant factor in determining the lubricant’s effective viscosity and, consequently, the bearing’s stable operating range. Unlike previous studies that rely on static volume fractions, these results provide a practical roadmap for engineers to optimize bearing performance by controlling aggregate packing fractions. Ultimately, this work offers a novel framework for designing next-generation, vibration-resistant hydrodynamic bearings for industrial turbomachinery. The key findings can be summarized as follows:

- Concave and wedge geometries outperform other forms. However, concave geometries have the highest critical stability number for a given eccentricity ratio. Longer bearings offer greater stability.
- Compared to a simple cylindrical bearing, concave bearing’s axial shape with maximum variation of $\Delta = 0.1$ and using a nanolubricant with $\Phi = 0.003\,\,and\,\,\beta = 7.77$, the relative increase in critical stability number starts with 13.32% at $\varepsilon_{o} = 0.2$ to 33.333% at $\varepsilon_{o} = 0.75$.
- The critical stability number increases with volume fraction and aggregate packing fraction.

## Supplementary Information

Supplementary Information.

## Data Availability

All data generated or analyzed during this study are included in this published article.

## List of symbols

$a$: Radii of primary nanoparticles, nm
$a_{a}$: Radii of aggregate nanoparticles, nm
c: Clearance in radial direction, m
$C_{fg}$: Friction parameter, the case of surface configuration
$C_{fn}$: Friction parameter, the case of nanoparticles addition
$C_{fgn}$: Friction parameter, combination of surface variation and nanoparticles addition
_D_: Journal minimum diameter, m
D^*^: Fractal index
e: Eccentricity, m
f: Coefficient of friction of the bearing
F: Frictional drag force, N
$F_{n}$: Force acting normal to the journal surface, N
$\overline{F}_{f}$: Friction force in dimensionless form, $\overline{F}_{f} = {{Fc} \mathord{\left/ {\vphantom {{Fc} {\mu_{bf} \,\omega R^{2} \,S_{o} }}} \right. \kern-0pt} {\mu_{bf} \,\omega R^{2} \,S_{o} }}$
$\overline{F}_{n}$: Normal force in dimensionless form, $\overline{F}_{n} = {{F_{n} } \mathord{\left/ {\vphantom {{F_{n} } {(\mu_{bf} \,\omega R^{3} \,S_{o} /c^{2} }}} \right. \kern-0pt} {(\mu_{bf} \,\omega R^{3} \,S_{o} /c^{2} }})$
h: Fluid film thickness in direction normal to journal axis, m
$\overline{h}$: Fluid film thickness in dimensionless form, $\overline{h} = {h \mathord{\left/ {\vphantom {h c}} \right. \kern-0pt} c}$
$\overline{p}$: Dimensionless film pressure, $\overline{p} = {p \mathord{\left/ {\vphantom {p {\left( {{{\mu_{bf} \,\omega \,R^{2} } \mathord{\left/ {\vphantom {{\mu_{bf} \,\omega \,R^{2} } {c^{2} }}} \right. \kern-0pt} {c^{2} }}} \right)}}} \right. \kern-0pt} {\left( {{{\mu_{bf} \,\omega \,R^{2} } \mathord{\left/ {\vphantom {{\mu_{bf} \,\omega \,R^{2} } {c^{2} }}} \right. \kern-0pt} {c^{2} }}} \right)}}$
R: Journal minimum radius, m
$S_{o}$: Bearing curvilinear length, m
t: Time, s
$$t_{o}$$: Characteristic time $$\left( {t_{o} = {1 \mathord{\left/ {\vphantom {1 \omega }} \right. \kern-0pt} \omega }} \right)$$, s
$\overline{t}$: Time in dimensionless form, $\overline{t} = {t \mathord{\left/ {\vphantom {t {t_{o} = \omega \,t}}} \right. \kern-0pt} {t_{o} = \omega \,t}}$
W: Radial load normal to the journal axis, N
$W^{*} ,\,W^{\prime}$: Steady load and perturbation load in dimensionless with respect to $(\mu_{bf} \,\omega \,R^{3} \,S_{o} /c^{2} )$
$W_{g}$: Load carrying capacity, surface variation scenario, N
$W_{n}$: Load carrying capacity, use of a nanofluid lubricant, N
$W_{g\,n}$: Load carrying capacity, combination of a nanofluid lubricant and surface variation, N
$\overline{W}$: Load-carrying capacity in dimensionless form, $\overline{W} = {W \mathord{\left/ {\vphantom {W {\left( {\mu_{bf} \,\omega \,R^{3} \,S_{o} /c^{2} } \right)}}} \right. \kern-0pt} {\left( {\mu_{bf} \,\omega \,R^{3} \,S_{o} /c^{2} } \right)}}$
$\overline{W}_{r} \,,\,\,\overline{W}_{t}$: Radial and transverse load components in dimensionless form with respect to $\,\left( {\mu_{bf} \,\omega \,R^{3} \,S_{o} /c^{2} } \right)$
(x, y, z): Cartesian co-ordinate system
$x,\,y^{\prime},\,z^{\prime}$: Coordinates along journal surface, normal and along journal axis respectively, m
Y, S: Coordinates normal to journal axis and along one side of the bearing surface respectively, m

## Greek symbols

β: Aggregate packing fraction, $\beta = {{a_{a} } \mathord{\left/ {\vphantom {{a_{a} } a}} \right. \kern-0pt} a}$
ε: Eccentricity ratio, $$\varepsilon = {e \mathord{\left/ {\vphantom {e c}} \right. \kern-0pt} c}$$
$\varepsilon_{o} ,\varepsilon^{\prime}$: Eccentricity ratios at equilibrium and perturbation, respectively
$\delta$: Maximum axial variation of the bearing geometry, m
$$\lambda$$: Stability number, $$\lambda = {{\mu_{bf} \,(R/c)^{3} \,S_{o} } \mathord{\left/ {\vphantom {{\mu_{bf} \,(R/c)^{3} \,S_{o} } {\omega \,M}}} \right. \kern-0pt} {\omega \,M}}$$
$\lambda_{cr}$: Critical stability number
$\Delta$: Dimensionless axial variation, $\Delta = {\delta \mathord{\left/ {\vphantom {\delta {S_{o} }}} \right. \kern-0pt} {S_{o} }}$
$\mu_{bf}$: Viscosity of plain engine oil, Pa s
$\mu_{nf}$: Viscosity of the Nano lubricant, Pa s
$\overline{\mu }$: Viscosity of nanolubricant in dimensionless form, $\overline{\mu } = {{\mu_{nf} } \mathord{\left/ {\vphantom {{\mu_{nf} } {\mu_{bf} }}} \right. \kern-0pt} {\mu_{bf} }}$
$\rho$: Fluid density, kg/m^3^
$\varphi$: Attitude angle, rad
$\Phi$: Nanoparticle volume fraction
$\phi_{o} \,,\,\phi^{\prime}$: Attitude angles for steady state and perturbation solutions, respectively
$\Phi_{m}$: Maximum packing volume fraction
$\theta$: Coordinate in tangential direction, rad
ω: Rotational angular velocity, rad/s

## References

[1] Kumar, A. & Mishra, S. S. Stability of a rigid rotor in turbulent hydrodynamic worn journal bearings. *Wear* **193**, 25–30 (1996).

[2] Ramesh, J., Majumdar, B. C. & Rao, N. S. Stability characteristics of rough submerged oil elliptical bearings under dynamic load. *Tribol. Int.* **30**(12), 857–863 (1997).

[3] Kakoty, S. K. & Majumdar, B. C. Effect of fluid inertia on stability of flow supported oil journal bearing: Linear perturbation analysis. *Tribol. Int.* **32**, 217–228 (1999).

[4] Raghunandana, K. & Majumdar, B. C. Stability of journal bearing systems using non-Newtonian lubricants: A non-linear transient analysis. *Tribol. Int.* **32**, 179–184 (1999).

[5] Weng, C. & Chen, C. Linear stability of short journal bearings with consideration of flow rheology and surface roughness. *Tribol. Int.* **34**, 507–516 (2001).

[6] Rahmani, F., Makki, E. & Giri, J. Influence of bearing wear on the stability and modal characteristics of a flexible rotor supported on powder-lubricated journal bearings. *Lubricants* **11**, 355. 10.3390/lubricants11090355 (2023).

[7] W. Hu, N. S. Feng and E. J. Hahn. Simulation and identification of rotor dynamic systems with multi-hydrodynamic bearings. *Australian MATLAB Users Conference*. (Crown Towers Melborne, 2000).

[8] Xiadong, Z., Xun, F., Huaqiang, S. & Zhengshui, H. Lubricating properties of Cyanex 302-modified MO S2 microspheres in base oil 500 SN. *Lubric. Sci.* **19**(1), 71–79 (2007).

[9] Liu, G. et al. Investigation of the mending effect and mechanism of copper nano-particles on a tribologically stressed surface. *Tribol. Lett.* **17**(4), 961–966 (2004).

[10] Tao, X., Jiazheng, Z. & Kang, X. The ball-bearing effect of diamond nanoparticles as an oil additive. *J. Phys. D Appl. Phys.* **29**, 2932–2937 (1996).

[11] Einstein, A. *Investigations on the Theory of the Brownian Movement* (Dover Publications Inc., 1956).

[12] Brinman, H. C. The viscosity of concentrated suspensions and solution. *New J. Phys.* **20**, 571–581 (1952).

[13] Batchelor, G. K. The effect of Brownian motion on the bulk stress in a suspension of spherical particles. *J. Fluid Mech.* **83**, 97–117 (1977).

[14] Bicerano, J., Douglas, J. F. & Brune, D. A. Model for the viscosity of particle dispersions. *J. Macromol. Sci. C Polym. Rev.* **39**(4), 561–642 (1999).

[15] Krieger, I. M. A mechanism for non-Newtonian flow in suspensions of rigid spheres. *Trans. Soc. Rheol.* **3**, 137–152 (1959).

[16] Kole, M. & Dey, T. K. Effect of aggregation on the viscosity of copper oxide-gear oil nanofluids. *Int. J. Therm. Sci.* **50**(9), 1741–1747 (2011).

[17] Chen, H., Ding, Y. & Tan, C. Reological behavior of nanofluids. *New J. Phys.* **9**(10), 367–367 (2007).

[18] Mahbubul, I. M., Saidur, R. & Amalina, M. A. Latest developments on the viscosity of nanofluids. *Int. J. Heat Mass Transfer* **55**, 874–885 (2012).

[19] Rudyak, V. Y. & Krasnolutskii, S. L. Dependence of the viscosity of nanofluids on nanoparticle size and material. *Phys. Lett.* **378**, 1845–1849 (2014).

[20] Nair, K. P., Ahmed, M. & Al-qahtani, S. T. Static and dynamic analysis of hydrodynamic journal bearing operating under nano-lubricants. *Int. J. Nanoparticles (IJNP)* **2**, 251–262 (2009).

[21] Shenoy, B. S., Binu, K. G., Pai, R., Rao, D. S. & Pai, R. S. Effect of nanoparticle additives on the performance of an externally adjustable fluid film bearing. *Tribol. Int.* **45**(1), 38–42 (2012).

[22] Binu, K. G., Shenoy, B. S., Rao, D. S. & Pai, R. A variable viscosity approach for the evaluation of load carrying capacity of oil lubricated journal bearing with TiO_2_ nanoparticles as lubricant additives. *Procedia Mater. Sci.* **6**, 1051–1067 (2014).

[23] Babu, K. S., Nair, K. P. & Rajendrakumar, P. K. Computational analysis of journal bearing operating under lubricant containing Al2O3 and ZnO nanoparticles. *Int. J. Eng. Sci. Technol.* **6**(1), 34–42 (2014).

[24] Rao, T. V. V. N., Rani, A. M. A., Sufian, S. & Mohamed, N. M. Thin film hydrodynamic bearing analysis using nanoparticle additive lubricant. *Eng. Appl. Nanotechnol.*10.1007/978-3-319-29761-3 (2017).

[25] Suryawanshi, S. R. & Pattiwar, J. T. Effect of TiO2 nanoparticles blended with lubricating oil on the tribological performance of the journal bearing. *Tribol. Ind.* **40**(3), 370–391 (2018).

[26] Wang, X. L., Zhu, K. Q. & Wen, S. Z. Thermohydrodynamic analysis of journal bearings lubricated with couple stress fluids. *Tribol. Int.* **34**, 335–343 (2001).

[27] Wang, X. L., Zhu, K. Q. & Gui, C. L. A study of a journal bearing lubricated by couple stress fluids considering thermal and cavitation effects. *J. Eng. Tribol.* **216**(Part J), 293–305 (2002).

[28] Guha, S. K. A theoretical analysis of dynamic characteristics of finite hydrodynamic journal bearings lubricated with couple stress fluids. *J. Eng. Tribol.* **218**, 125–133 (2004).

[29] Crosby, W. A. & Chetti, B. The static and dynamic characteristics of a two-lobe journal bearing lubricated with couple-stress fluid. *Tribol. Trans.* **52**, 262–268 (2009).

[30] Mokhiamer, U. A., Crosby, W. A. & El-Gamal, H. A. Study of a journal bearing lubricated by fluids with couple stress considering the elasticity of the liner. *Wear* **224**, 194–201 (1999).

[31] Ibhadode, A. O. Elastohydrodynamic analysis of an offsed journal bearing lubricated with couple stress fluid. *Int. J. Eng. Res. Afr.* **2**, 53–62 (2010).

[32] Dass, T., Gunakala, S. R. & Comissiong, D. M. G. The combined effect of couple stress, variable viscosity and velocity-slip on the lubrication of finite journal bearings. *Ain Shams Eng. J.* **11**, 501–5018 (2020).

[33] Zhu, J., Qian, H., Wen, H., Zeng, L. & Zhu, H. Analysis of misaligned journal bearing lubrication performance considering the effect of lubricant couple stress and shear thinning. *J. Mechan.* **37**, 282–290 (2021).

[34] Senatore, A., Ruggiero, A., Jevremovi´c, V. & Nedeff, V. Effects of couple stresses on the unsteady performance of finite lubricated bearings. *J. Mech. Eng.* **55**, 141–147 (2009).

[35] Mehta, N. P., Rattan, S. S. & Verma, R. Stability analysis of two-lobe hydrodynamic journal bearing with couple stress lubricant. *J. Eng. Appl. Sci.* **5**(1), 69–74 (2010).

[36] Kumar, V., Lambha, S. K. & Verma, R. Chaotic study of dynamic stability in spindle shaft using couple stress fluid as lubricant. *Int. J. Adv. Mechanical Civil Eng.* **4**(3), 21–25 (2017).

[37] Hamed, O. & Saber, E. Stability analysis of hydrodynamic journal bearings of finite length. *PSERJ* **8**, 236–251 (2004).

[38] Saber, E. & Abdou, K. M. Effect of rotor misalignment on stability of journal bearings with finite length. *Alex. Eng. J.* **59**, 3407–3417. 10.1016/j.aej.2020.05.020 (2020).

[39] Awad, H., Abdou, K. M. & Saber, E. Steady state characteristics and stability limits of oil lubricated journal bearings using Titanium Dioxide nanoparticles as lubricant additives. *Res. Eng.* **20**, 101486. 10.1016/j.rineng.2023.101486 (2023). 10.1038/s41598-025-97948-7 PMC12053646 40325062

[40] Awad, H., Abdou, K. M. & Saber, E. The effect of axial geometrical variations on the steady state characteristics of oil lubricated journal bearings using titanium dioxide nanoparticles as lubricant. *Sci. Rep.* **15**, 15701. 10.1038/s41598-025-97948-7 (2025). 40325062 10.1038/s41598-025-97948-7 PMC12053646

[41] Li, X. et al. Dynamic modeling of a spline-shaft system including time varying fretting friction. *MSSP* **249**, 114058. 10.1016/j.ymssp.2026.114058 (2026).

[42] Li, X. et al. Dynamic modeling and analysis of a shaft system with the floating spline and angular misalignment. *Mech. Syst. Signal Process.* **249**, 114058. 10.1016/j.ymssp.2026.114058 (2026).

[43] Li, X. et al. Dynamic modlling and vibration analysis of parallel misalignment shaft system considering the spline time-varying meshing point. *MSSP* **241**, 113425. 10.1016/j.ymssp.2025.113425 (2025).

[44] Rahman, M. A. et al. Review on Nanofluids: Preparation, properties, stability, and thermal performance augmentation in heat transfer applications. *ACS Omega* **9**(30), 32328–32349. 10.1021/acsomega.4c03279 (2024). 39100289 10.1021/acsomega.4c03279 PMC11292633

[45] Bawa’neh, H., Albiss, B. A. & Ocak, Y. S. Improving tribological performance of lubricating oil using functionalized nanodiamonds as an additive material. *RSC Adv.* **15**, 26766–26775. 10.1039/D5RA03156G (2025). 40740217 10.1039/d5ra03156g PMC12308276

[46] Bondarenko, R., Bukichev, Y., Dzhaga, A., Dzhardimalieva, G. & Solyaev, Y. Micropolar effects on the effective shear viscosity of nanofluids. *Phys. Fluids* **36**, 062004. 10.1063/5.0208850 (2024).

[47] Mishra, S. & Aggarwal, S. A critical review of the effect of nano-lubricant on the performance of hydrodynamic journal bearing. *J. Tribol.*10.30678/fjt.127785 (2023).

