# Supplementary material for: Stability analysis of hydrodynamic journal bearings with variable axial geometrical configuration using titanium dioxide nanoparticles as lubricant additives
Source: Sci Rep. 2026 Apr 24;16:13389. doi: 10.1038/s41598-026-47711-3 (PMC13109420; doi:10.1038/s41598-026-47711-3)
Supplement: Supplementary file 1 — Supplementary Information. [file 41598_2026_47711_MOESM1_ESM.docx]

**Name of the Journal:** Scientific Reports<srep@nature.com>

Manuscript Number: Ref: Submission ID 8d744a19-88b8-4256-8920-84e76814bb0d

# Title of the Paper: "Stability Analysis of Hydrodynamic Journal Bearings with Variable Axial Geometrical Configuration Using Titanium Dioxide Nanoparticles as Lubricant Additives"

I'd like to submit a reply to our work titled “Stability Analysis of Hydrodynamic Journal Bearings with Variable Axial Geometrical Configuration Using Titanium Dioxide Nanoparticles as Lubricant Additives” that addresses all reviewers' comments.

Responses to each reviewer's comments are listed below:

Comments on “Stability Analysis of Hydrodynamic Journal Bearings with Variable Axial Geometrical Configuration Using Titanium Dioxide Nanoparticles as Lubricant Additives”

**Reviewer #1**

| No. | Comment | Reply (Response) |
| --- | --- | --- |
| 1 | The introduction lacks a clear justification for selecting TiO2 nanoparticles over other commonly used nanoparticles (e.g., Al2O3, MoS2). | **Preface:**  TiO2 was selected primarily because the present formulation adopts a viscosity model calibrated with published TiO2/SAE30 experimental data and because the current work is intended as a dynamic-stability extension of our previous TiO2-based journal-bearing studies. We have revised the Introduction to make clear that the choice of TiO2 reflects model/data consistency and continuity with prior work, rather than a claim that TiO2 is universally superior to all other nanoparticles. We also added a short comparative discussion of commonly used nanolubricant additives such as Al2O3 and MoS2 and explicitly stated that a cross-material comparison is outside the present scope.  **Response:**  TiO2 was used because TiO2/SAE30 experimental rheology data previously used in the research [22, 39, and 40] confirm the modified Krieger–Dougherty formulation proposed here. The current article builds on the authors' previous TiO2-based static/stability studies, therefore using the same nanoparticle isolates the effects of shape and aggregation. TiO2 is not offered as the "best"; however, it is a representative and data-supported case study.  At the end of the introduction section, the following text is added in the manuscript  **Text in the manuscript:**  **Identification of the Research Gap and Current Objectives:** Building upon these previous analyses, the current study aims to continue the detailed investigation suggested by Refs. [39] and [40] by extending the scope into the dynamic and stability domains. To the best of the authors' knowledge, no prior literature has considered the stability analysis of hydrodynamic journal bearings featuring variable axial geometrical configurations. This effort seeks to address this critical gap by assessing how changes in lubricant viscosity, driven by nanoparticle concentration, size, and aggregation, influence the stability of bearings with varying axial shapes. TiO₂ is utilized as a representative, data-supported case study to isolate the specific effects of shape and aggregation on stability limitations and dynamic conduct. |
| 2 | Recent literature on nanoparticle aggregation and its effect on lubricant rheology is not sufficiently covered. | **Response:**  Insert a paragraph just before introducing the equations for the modified Krieger-Dougherty model.  **Text in the manuscript:**  **Viscosity model $\left(\bar{\mu}\right)()$**  While the experimental protocols for TiO₂ nanolubricant preparation and baseline viscosity measurements follow the established methodology of Ref. [22], recent industrial reviews emphasize that the rheological behavior of nanofluids is fundamentally dictated by the stability and aggregation state of the suspension [44, 45]. Modern research confirms that nanoparticles naturally form clusters due to high surface energy, effectively trapping a portion of the base oil within the aggregate structure. This phenomenon increases the effective volume fraction of the solid phase, directly supporting the use of the aggregate packing fraction and the ratio of aggregate-to-primary particle size [46]. Unlike classical models that assume non-interacting spheres, these recent reviews highlight that the aggregation degree is the primary driver of non-Newtonian viscosity shifts and enhanced load-carrying capacity in hydrodynamic bearings [47]. Consequently, the modified Krieger–Dougherty formulation is employed here to provide a physically accurate representation of the lubricant’s resistance to shear in high-speed applications  The modified Krieger-Dougherty viscosity model may be applied in this study as [15],    **Added references:**  [41] X. Li, J. Liu, Jing Liu, Y. Xu, G. Pan, Z. Shi, dynamic modeling of a spline-shaft system including time varying fretting friction, MSSP [Volume 249](https://www.sciencedirect.com/journal/mechanical-systems-and-signal-processing/vol/249/suppl/C), 1 April 2026, 114058. <https://doi.org/10.1016/j.ymssp.2026.114058> [42] X. Li, J. Liu, Jing Liu, Y. Xu, G. Pan, Z. Shi, Dynamic modeling and analysis of a shaft system with the floating spline and angular misalignment, [Volume 249](https://www.sciencedirect.com/journal/mechanical-systems-and-signal-processing/vol/249/suppl/C), 1 April 2026, 114058. <https://doi.org/10.1016/j.ymssp.2026.114058> [43] X. Li, J. Liu, Jing Liu, Y. Xu, , G. Pan, Z. Shi, Dynamic modlling and vibration analysis of parallel misalignment shaft system considering the spline time-varying meshing point, MSSP [Volume 241](https://www.sciencedirect.com/journal/mechanical-systems-and-signal-processing/vol/241/suppl/C), 1 December 2025, 113425. <https://doi.org/10.1016/j.ymssp.2025.113425>  [44] [M. A. Rahman](https://pubmed.ncbi.nlm.nih.gov/?term=%22Rahman%20MA%22%5bAuthor%5d), [S. M. Hasnain](https://pubmed.ncbi.nlm.nih.gov/?term=%22Hasnain%20SMM%22%5bAuthor%5d), [S. Pandey](https://pubmed.ncbi.nlm.nih.gov/?term=%22Pandey%20S%22%5bAuthor%5d), [A. Tapalova](https://pubmed.ncbi.nlm.nih.gov/?term=%22Tapalova%20A%22%5bAuthor%5d), [N. Akylbekov](https://pubmed.ncbi.nlm.nih.gov/?term=%22Akylbekov%20N%22%5bAuthor%5d), [R. Zairov](https://pubmed.ncbi.nlm.nih.gov/?term=%22Zairov%20R%22%5bAuthor%5d)., Review on Nanofluids: Preparation, Properties, Stability, and Thermal Performance Augmentation in Heat Transfer Applications, ACS Omega, Jul 15; 9(30):32328–32349. Doi: [10.1021/acsomega.4c03279](https://doi.org/10.1021/acsomega.4c03279) . [45] H. Bawa'neh, B. A. Albiss and Y. S. Ocak, Improving tribological performance of lubricating oil using functionalized nanodiamonds as an additive material. [*RSC Adv.*](https://doi.org/10.1039/2046-2069/2011), 2025, ****15****, 26766-26775. DOI: [10.1039/D5RA03156G](https://doi.org/10.1039/D5RA03156G) [46] [R. Bondarenko](javascript:;), [Yu. Bukichev](javascript:;) , [A. Dzhaga](javascript:;), [G. Dzhardimalieva](javascript:;), [Y. Solyaev](javascript:;), Micropolar effects on the effective shear viscosity of nanofluids, Physics of Fluids 36, 062004 (2024), [doi.org/10.1063/5.0208850](https://doi.org/10.1063/5.0208850)  [47] [S. Mishra](https://www.researchgate.net/scientific-contributions/Santwna-Mishra-2269575236?_sg%5B0%5D=Z-6-4_q08ZJlSKeXsMVV5QW-RKUaP_lKiJ8N5yhnvF-UhLuflWJ0FU4UELN849S0GqYb5vU.sNGX-XAFaY2_-0gWjciaObLC7R956b9yYW34xHk93zJbdClOo2SlKDHKHRk2DlGzTuU_X3rkEaxnH-4BlYVKxA&_sg%5B1%5D=V8u4pt1FZo2IK20SjvMBUkmiD2PIUu8ykqM9WCd6iJZbVY97UroPdsFQ4tIeLb0AhuevDwU.Jlz535wFexnTM7xgkxHP4KOHTbToRSRrWUYMfeyEeACfHeS-MjwYA0mwWRWCpVVVKUVARO26RGbNa8-jKKGPSA&_tp=eyJjb250ZXh0Ijp7ImZpcnN0UGFnZSI6InB1YmxpY2F0aW9uIiwicGFnZSI6InB1YmxpY2F0aW9uIiwicG9zaXRpb24iOiJwYWdlSGVhZGVyIn19) and [S. Aggarwal](https://www.researchgate.net/profile/Shipra-Aggarwal-3?_sg%5B0%5D=Z-6-4_q08ZJlSKeXsMVV5QW-RKUaP_lKiJ8N5yhnvF-UhLuflWJ0FU4UELN849S0GqYb5vU.sNGX-XAFaY2_-0gWjciaObLC7R956b9yYW34xHk93zJbdClOo2SlKDHKHRk2DlGzTuU_X3rkEaxnH-4BlYVKxA&_sg%5B1%5D=V8u4pt1FZo2IK20SjvMBUkmiD2PIUu8ykqM9WCd6iJZbVY97UroPdsFQ4tIeLb0AhuevDwU.Jlz535wFexnTM7xgkxHP4KOHTbToRSRrWUYMfeyEeACfHeS-MjwYA0mwWRWCpVVVKUVARO26RGbNa8-jKKGPSA&_tp=eyJjb250ZXh0Ijp7ImZpcnN0UGFnZSI6InB1YmxpY2F0aW9uIiwicGFnZSI6InB1YmxpY2F0aW9uIiwicG9zaXRpb24iOiJwYWdlSGVhZGVyIn19), A critical review of the effect of nano-lubricant on the performance of hydrodynamic journal bearing, Journal of Tribology 40(4), 2023. DOI: [10.30678/fjt.127785](https://doi.org/10.30678/fjt.127785) |
| 3 | The boundary condition for cavitation (negative pressure set to zero) is mentioned but not clearly defined. | **Preface:**  To provide clear description of the cavitation boundary condition, a paragraph is added to the Numerical Formulation or Governing Equations section. This paragraph specifies both the mathematical conditions and the physical location of the film rupture. To account for fluid film rupture in the bearing's divergent regions, the Reynolds boundary condition (also known as the Swift-Stieber condition) is used.  **Text in the manuscript:**  To account for the physical occurrence of film rupture in the diverging region of the bearing, the Reynolds boundary condition is used. In this technique, the pressure distribution is solved by converting any expected sub-atmospheric (negative) pressures to ambient (known as the Swift-Stieber condition), both the pressure and its gradient vanish simultaneously. Applying the Swift-Stieber condition, the boundary conditions for the pressure variable $\bar{p}$ may be written as follows,   |
| 4 | The conclusions are general and do not offer specific design recommendations or practical insights | **Preface:**  This study provides a comprehensive stability analysis of hydrodynamic journal bearings featuring variable axial geometries and TiO₂ nanofluid lubrication. By integrating the modified Krieger–Dougherty viscosity model with a dynamic vibration simulation, the following specific design recommendations and practical insights are established:   - **Optimal Geometry Selection:** For high-speed applications where stability is the primary concern, the **concave axial configuration** is the superior design choice. It consistently outperforms conical, convex, and wavy geometries by offering the highest stability threshold across all tested eccentricity ratios. - **Nanoparticle Concentration Limits:** While increasing the volume fraction ($\Phi$) of TiO₂ nanoparticles enhances the load-carrying capacity, engineers should limit the concentration to a range that prevents excessive aggregate-induced viscosity, which can lead to increased power loss.. - **Impact of Aggregation on Stability:** The research reveals that the **aggregate packing fraction** is a critical design variable. As nanoparticle clusters grow, the effective viscosity rises, shifting the stability map toward higher stability numbers. Designers must ensure stable dispersion to avoid uncontrolled aggregation, which may lead to unpredictable dynamic behavior at high eccentricity. - **Geometric Synergy:** A significant practical insight is the synergistic effect between **concave geometry and nanofluid additives**. The concave shape compensates for the potential reduction in film thickness caused by the presence of nanoparticle aggregates, ensuring a safer operating margin than standard cylindrical bearings.   In summary, this work provides a robust decision-making roadmap for engineers. By selecting a **concave axial profile** and utilizing a **stabilized TiO₂ nanolubricant**, industrial rotors can achieve significantly higher stability thresholds, allowing for safer operation at higher speeds and loads than previously possible with conventional bearing designs.  **Text in the manuscript:**  Conclusions  The current study looks at how adopting an axial geometrical design for hydrodynamic bearings lubricated with nanolubricant comprising titanium dioxide nanoparticles as lubricant additives affects the bearing's dynamic performance and stability limits. A curvilinear coordinate system is used to generate the Reynolds-like equation that determines pressure within the bearings. The governing equations for dynamic settings are derived using a perturbation technique. This research demonstrates that the dynamic stability of high-speed rotors can be significantly enhanced through the strategic combination of axial bearing profiling and nanofluid additives. The primary finding identifies the concave axial configuration as the optimal design choice, consistently providing a superior stability threshold compared to conical, convex, or wavy geometries.  Furthermore, by utilizing a modified Krieger-Dougherty model, this study proves that nanoparticle aggregation is a dominant factor in determining the lubricant’s effective viscosity and, consequently, the bearing’s stable operating range. Unlike previous studies that rely on static volume fractions, these results provide a practical roadmap for engineers to optimize bearing performance by controlling aggregate packing fractions. Ultimately, this work offers a novel framework for designing next-generation, vibration-resistant hydrodynamic bearings for industrial turbomachinery. The key findings can be summarized as follows:   - Concave and wedge geometries outperform other forms. However, concave geometries have the highest critical stability number for a given eccentricity ratio. Longer bearings offer greater stability. - Compared to a simple cylindrical bearing, concave bearing’s axial shape with maximum variation of $\Delta=0.1$ and using a nanolubricant with $\Phi=0.003 and \beta=7.77$, the relative increase in critical stability number starts with 13.32% at $\varepsilon_{o}=0.2$ to 33.333% at $\varepsilon_{o}=0.75$. - The critical stability number increases with volume fraction and aggregate packing fraction. |
| 5 | The effect of aggregate packing fraction is presented, but its interaction with volume fraction and geometry is not analyzed in depth. | We thank the reviewer for this valuable suggestion. In the revised manuscript, we expanded the discussion of aggregate packing fraction by comparing its effect at different nanoparticle volume fractions and by clarifying how this interaction modifies the stability response for the selected bearing geometries.  **Response:**  **Text in the manuscript:**  Figure (10a) depicts the effect of the aggregate packing fraction ($\beta$) on the critical stability number ($\lambda_{cr}$). While it is clear that a higher packing fraction raises the stability threshold for all eccentricity ratios, the data also show a substantial interaction effect with the bearing's axial shape. The concave form is far more sensitive to changes in ($\beta$) than the convex or wavy surfaces. This shows that the concave geometry's higher center film thickness acts as a 'buffer', allowing the increased effective viscosity resulting from high aggregate packing to be translated into damping energy more efficiently while avoiding premature film rupture. Additionally, the interaction with nanoparticle volume fraction ($\Phi$) is non-linear; at low volume concentrations, the geometric profile has a greater influence than the highest value of volume fraction concentration. However, the aggregation state takes over as the primary driver of stability when the volume percentage rises, hence reducing the performance difference between the various axial forms. This suggests that maintaining a stable operating margin for severely loaded bearings depends more on controlling nanoparticle stability and cluster size than it does on physically modifying the axial profile. The effect of bearing length on the value of the critical stability number is illustrated in Fig. 10b. as the bearing length increases the critical stability number increases. |

**Reviewer #2:**

This work investigated the dynamic behavior of fluid film bearings using various axial geometrical configurations and nanofluid lubrication, which can provide guidance for the design of hydrodynamic journal bearings. The subject of the manuscript is interesting. However, the current version of the paper is still needed substantial improved. My suggestions are as follows:

| No. | Comment | Reply (Response) |
| --- | --- | --- |
| 1 | The literature review section lacks logical structure. The first paragraph is nearly two pages long, poorly organized, and difficult to follow. I suggest splitting it into several shorter, logically coherent paragraphs. | We agree. The Introduction has been restructured into shorter thematic paragraphs to improve readability and logical flow. Specifically, the revised version now separates prior work on hydrodynamic journal-bearing stability, nanolubricant applications in journal bearings, rheology/aggregation models, and related rotor-system dynamic studies.  **Text in the manuscript:**  **Introduction**  **Foundations of Journal Bearing Stability:** The stability analysis of journal bearings rotating at high speeds is of critical importance to modern rotor dynamics. Due to the inherent relationship between fluid film dynamics and rotor performance, researchers have explored various factors influencing system behavior. Kumar and Mishra [1] numerically investigated the impact of geometric changes due to wear following turbulent lubrication theory. Rameshet et al. [2] examined the effect of surface roughness on the stability of submerged oil elliptical journal bearings under dynamic loads using an average flow model. Furthermore, Kakoty and Majumdar [3] utilized a linear perturbation approach to study fluid film inertia on the stability of bearings mounted on flexible supports, finding that inertia effects are vital for reliable predictions in high-speed utilities.  **Rheological Considerations and Computational Modeling:** Advanced lubrication research has expanded to address complex rheological behaviors and modeling techniques. Raghunandana and Majumdar [4] investigated the influence of non-Newtonian lubricant behavior caused by polymers on bearing stability. Similarly, Weng and Chen [5] studied linear stability by accounting for surface roughness and flow rheology, linearizing the modified Reynolds equation and rotor motion equations around an equilibrium location. The effects of wear on the dynamic behavior of flexible rotors supported by powder-lubricated bearings were explored by Rahmani et al. [6]. To facilitate these analyses, Hu et al. [7] developed modeling software using Matlab and Simulink to simulate and identify dynamic behaviors in rotors supported by multiple hydrodynamic bearings.  **Nanoparticles as Lubricant Additives:** The integration of nanoparticles as lubricant additives represents a significant advancement in enhancing bearing performance. Beyond improving lubrication, nanoparticles can be used to coat friction surfaces with a protective layer [8]. These additive depositions have been shown to compensate for material loss through indirect effects [9] and effectively lower friction surface roughness [10]. While nanofluids exhibit higher effective viscosity than conventional base fluids—determined primarily by concentration and size—there remains a scarcity of data regarding their impact on dynamic stability. Although many classical models for nanofluid viscosity have been established in the literature [11–19], most research has concentrated on the steady-state properties of bearings operating with various nanoparticle additives [20–25].  **Impact of Couple Stress Fluids on Dynamic Stability:** The properties of journal bearings lubricated by couple stress fluids have also been extensively documented [26–33]. Findings generally suggest that utilizing couple stress lubricants improves load-carrying capacity and reduces the coefficient of friction. Senator et al. [34] investigated the effect of couple stress fluid characteristics on film forces specifically during unstable operation situations. Mehta et al. [35] utilized a finite element approach to study the stability of two-lobe hydrodynamic bearings, revealing that the couple stress parameter significantly impacts stiffness and damping coefficients while increasing the stability threshold speed. Additionally, Kumar et al. [36] analyzed the dynamic behavior of spindle motion in lathe machines, solving non-linear equations of motion via the Runge-Kutta approach to confirm that couple stress fluids enhance overall rotor stability.  **Previous Findings on Misalignment and Axial Geometry:** Foundational research by Hamed and Saber [37] utilized perturbation techniques and numerical simulations of vibration behavior to evaluate rotor stability under dynamic conditions. Complementary studies by Saber and Abdou [38] provided a thorough investigation into the dynamic stability and responses of misaligned fluid film bearings. Recently, Awad et al. [39] specifically examined the impact of titanium dioxide (TiO₂) nanoparticle volume percentages and aggregate sizes on the steady-state and stability constraints of plain journal bearings. Subsequent work [40] investigated the influence of axial geometrical arrangements—including conical (wedge), concave, convex, and wavy surfaces—on steady-state characteristics. This research established that modifying the bearing’s axial shape increases load-carrying capacity and decreases friction compared to standard cylindrical designs, with concave geometry demonstrating clear superiority.  **Contemporary Developments in Rotor System Dynamics:** While the current study focuses on the localized fluid film dynamics and stability of specifically profiled journal bearings, it is important to situate these findings within the broader context of complex rotor system assemblies. Recent literature in rotor dynamics has increasingly focused on system-level vibration monitoring and fault identification [41]. Advances in modeling nonlinear behaviors and bifurcation in rotating shafts [42] highlight the sensitivity of the entire assembly to small changes in support conditions. By providing a precise stability map for non-conventional bearing geometries and aggregated nanolubricants, the present work offers the high-fidelity input data required for these comprehensive rotor-system diagnostic frameworks [43].  **Identification of the Research Gap and Current Objectives:** Building upon these previous analyses, the current study aims to continue the detailed investigation suggested by Refs. [39] and [40] by extending the scope into the dynamic and stability domains. To the best of the authors' knowledge, no prior literature has considered the stability analysis of hydrodynamic journal bearings featuring variable axial geometrical configurations. This effort seeks to address this critical gap by assessing how changes in lubricant viscosity, driven by nanoparticle concentration, size, and aggregation, influence the stability of bearings with varying axial shapes. TiO₂ is utilized as a representative, data-supported case study to isolate the specific effects of shape and aggregation on stability limitations and dynamic conduct.  The novelty of the present study lies in its integrated dynamic-stability analysis of hydrodynamic journal bearings with variable axial geometrical configurations operating with a TiO2-based nanolubricant while explicitly accounting for nanoparticle aggregation effects on lubricant rheology. Although previous studies have separately examined the stability of conventional journal bearings, the steady-state performance of nanolubricated bearings, and the static behavior of axially modified bearing geometries, the combined influence of axial geometry variation, nanoparticle concentration, and aggregate-induced viscosity modification on the stability limits and nonlinear dynamic response of journal bearings has not been adequately addressed. In this context, the present work extends earlier investigations on plain TiO2-lubricated journal bearings and axially varying geometries by incorporating a modified Krieger-Dougherty viscosity model into a Reynolds-type formulation in curvilinear coordinates and by evaluating the resulting stability thresholds through perturbation analysis and time-domain simulation. This combined framework enables a more realistic assessment of how bearing geometry and aggregation-sensitive nanolubricant behavior interact to govern the transition from stable to unstable operation, thereby providing a more comprehensive basis for the design and performance enhancement of advanced hydrodynamic journal bearings. |
| 2 | The manuscript subsequently establishes a dynamic model of the rotor system and analyzes its stability. However, the literature review does not include a comprehensive overview of studies related to the rotor system. Below are several references that may be helpful for your revision. https://doi.org/10.1016/j.ymssp.2026.114058; https://doi.org/10.1016/j.ijmecsci.2025.110192; https://doi.org/10.1016/j.engstruct.2025.120773; https://doi.org/10.1016/j.ymssp.2025.112363; https://doi.org/10.1016/j.jsv.2024.118612; https://doi.org/10.1016/j.engfailanal.2026.110559; https://doi.org/10.1016/j.ymssp.2025.113425. | By defining them as the "system-level" environment into which our particular "bearing-level" research fits, authors can incorporate a selection of these (e.g., 2 or 3) to guarantee the state-of-the-art in the broader engineering discipline.  **Text in the manuscript:**  **Contemporary Developments in Rotor System Dynamics:** While the current study focuses on the localized fluid film dynamics and stability of specifically profiled journal bearings, it is important to situate these findings within the broader context of complex rotor system assemblies. Recent literature in rotor dynamics has increasingly focused on system-level vibration monitoring and fault identification [41]. Advances in modeling nonlinear behaviors and bifurcation in rotating shafts [42] highlight the sensitivity of the entire assembly to small changes in support conditions. By providing a precise stability map for non-conventional bearing geometries and aggregated nanolubricants, the present work offers the high-fidelity input data required for these comprehensive rotor-system diagnostic frameworks [43]. |
| 3 | It is recommended that a paragraph explaining the innovation of this manuscript be added after the introduction. | To highlight the innovation of the present work effectively, this paragraph be placed immediately following the literature review and just before the "Problem Formulation" or "Materials and Methods" section.  **Text in the manuscript:**  The novelty of the present study lies in its integrated dynamic-stability analysis of hydrodynamic journal bearings with variable axial geometrical configurations operating with a TiO2-based nanolubricant while explicitly accounting for nanoparticle aggregation effects on lubricant rheology. Although previous studies have separately examined the stability of conventional journal bearings, the steady-state performance of nanolubricated bearings, and the static behavior of axially modified bearing geometries, the combined influence of axial geometry variation, nanoparticle concentration, and aggregate-induced viscosity modification on the stability limits and nonlinear dynamic response of journal bearings has not been adequately addressed. In this context, the present work extends earlier investigations on plain TiO2-lubricated journal bearings and axially varying geometries by incorporating a modified Krieger-Dougherty viscosity model into a Reynolds-type formulation in curvilinear coordinates and by evaluating the resulting stability thresholds through perturbation analysis and time-domain simulation. This combined framework enables a more realistic assessment of how bearing geometry and aggregation-sensitive nanolubricant behavior interact to govern the transition from stable to unstable operation, thereby providing a more comprehensive basis for the design and performance enhancement of advanced hydrodynamic journal bearings. |
| 4 | Some symbols in the manuscript have not been defined or explained. | We thank the reviewer for noting this issue. The manuscript has been carefully revised to define all symbols at first appearance and to ensure consistency between the equations, figure captions, and nomenclature list. |
| 5 | Figure 8(a) is incomplete. | We appreciate the reviewer’s careful reading. Figure 8 has been checked in the revised manuscript to ensure that all subfigures are complete, legible, and properly labeled. |
| 6 | The captions are difficult to understand, for example, the caption of Figure 8. | We agree and have revised the figure captions throughout the manuscript to make them self-contained and easier to understand. In particular, the caption of Figure 8 now clearly identifies the operating condition, parameter values, and the meaning of each subfigure corresponding to stable, critical, and unstable responses. |

The current abstract has been rewritten to better reflect the changes we made to the Introduction and Conclusion;

**Abstract**

The dynamic stability of high-speed rotor systems is highly dependent on the interaction between bearing geometry and lubricant rheology. The present work examines the stability of hydrodynamic journal bearings with four different axial configurations: conical (wedge), concave, convex, and wavy, lubricated with TiO₂ nanofluids. The integration of a modified Krieger-Dougherty viscosity model that takes into consideration packing fractions and nanoparticle aggregation, extending beyond conventional static volume-fraction assumptions, is an important advance of this work. The governing Reynolds-like equation is expressed in curvilinear coordinates and numerically solved to determine the transition between stable and unstable vibrations. The results show that nanoparticle aggregate size and packing percent strongly influence the critical stability number, with larger aggregation generally improving the stable working range. The stability limitations are studied using a numerical simulation of the journal's vibration behavior. Vibration data is collected to create a stability map, which represents the transition between stable and unstable vibrations as a function of eccentricity ratio and stability number. It is found that concave and wedge geometries outperform the other shapes investigated; but the concave geometry is recommended to attain the highest critical stability number for a given eccentricity ratio.
